# Deregulated expression of miR-29a-3p, miR-494-3p and miR-660-5p affects sensitivity to tyrosine kinase inhibitors in CML leukemic stem cells

**DOI:** 10.18632/oncotarget.17706

**Published:** 2017-05-08

**Authors:** Simona Salati, Valentina Salvestrini, Chiara Carretta, Elena Genovese, Sebastiano Rontauroli, Roberta Zini, Chiara Rossi, Samantha Ruberti, Elisa Bianchi, Greta Barbieri, Antonio Curti, Fausto Castagnetti, Gabriele Gugliotta, Gianantonio Rosti, Micaela Bergamaschi, Agostino Tafuri, Enrico Tagliafico, Roberto Lemoli, Rossella Manfredini

**Affiliations:** ^1^ Center for Regenerative Medicine, University of Modena and Reggio Emilia, Modena, Italy; ^2^ Department of Experimental, Diagnostic and Specialty Medicine, Institute of Hematology “L. and A. Seràgnoli”, University of Bologna, S.Orsola-Malpighi Hospital, Bologna, Italy; ^3^ Department of Internal Medicine (DiMI), Clinic of Hematology, University of Genoa, IRCCS Azienda Ospedaliera Universitaria S. Martino-IST, Genoa, Italy; ^4^ Department of Clinical and Molecular Medicine, Clinic of Hematology, University “La Sapienza”, Rome, Italy; ^5^ Center for Genome Research, University of Modena and Reggio Emilia, Modena, Italy

**Keywords:** chronic myeloid leukemia, leukemic stem cells, tyrosine kinase inhibitors, microRNAs, apoptosis

## Abstract

The development of Imatinib mesylate (IM), which targets the oncogenic BCR-ABL fusion protein, has greatly improved the outcome of Chronic Myeloid Leukemia (CML) patients. However, BCR-ABL–positive progenitors can be detected in CML patients in complete cytogenetic response. Several evidence suggests that CML stem cells are intrinsically resistant to Tyrosine Kinase Inhibitors (TKI), and therefore they represent the most likely candidate responsible for disease relapse.

In this work, we investigated the microRNA (miRNA) expression profile of different subpopulations of CML Leukemic Stem Cells (LSCs): Lin-CD34+CD38- and Lin-CD34-CD38- cells. These cell fractions have been previously shown to be endowed with TKI intrinsic resistance. Our analysis identified 33 common deregulated miRNAs in CML LSCs. Among those, 8 miRNAs were deregulated in CML independently from BCR-ABL kinase activity and therefore are likely to be involved in the BCR-ABL-independent resistance to TKI that characterizes CML LSCs. In particular, the up-regulation of miR-29a-3p and miR-660-5p observed in CML LSCs, led to the down-regulation of their respective targets *TET2* and *EPAS1* and conferred TKI-resistance to CML LSCs *in vitro*. On the other hand, miR-494-3p down-regulation in CML LSCs, leading to *c-MYC* up-regulation, was able to decrease TKI-induced apoptosis. These results demonstrate that aberrant miRNA expression in CML LSCs could contribute to the intrinsic TKI-resistance observed in these cell populations, and support the development of novel therapies aimed at targeting aberrantly regulated miRNAs or their targets in order to effectively eradicate CML LSCs.

## INTRODUCTION

Chronic Myeloid Leukemia (CML) is a stem cell-derived malignant disorder characterized by the reciprocal translocation t(9;22) leading to the BCR-ABL fusion gene. The encoded 210 KDa protein has constitutively elevated tyrosine kinase activity and drives Hematopoietic Stem Cells (HSCs) transformation [1]. The BCR-ABL Tyrosine Kinase Inhibitors (TKIs), such as Imatinib mesylate, are the standard therapy for CML patients. Imatinib has been shown to induce complete molecular response in more than 80% of newly diagnosed patients in chronic phase [2]. However, it has been unable to completely eliminate BCR-ABL-expressing leukemic cells [3]. In order to achieve durable remission, CML patients require lifelong treatment with TKIs, at considerable cost and despite harmful side effects. There is an increasing body of evidence suggesting that TKIs are not effective in killing leukemic stem cells (LSCs) in CML [4, 5]. In particular, Corbin *et al*. demonstrated that CML LSCs survive IM treatment independently of BCR-ABL kinase activity [4]. Recently, our group identified a novel quiescent LSCs subset (Lin-CD34-CD38-), endowed with stem cell properties and intrinsically resistant to TKI treatment both *in vitro* and *in vivo* [6]. It is therefore clear that a definitive treatment for CML requires the elimination of LSCs. Thus, gaining further understanding on the molecular and functional properties of the stem cell compartment in CML is mandatory for the development of more effective therapies that will eliminate TKI-resistant LSCs.

MicroRNAs (miRNAs) are small non-coding RNAs that control gene expression and play an important role in several biological processes such as differentiation [7], proliferation [8], and apoptosis [9]. In the last few years, increasing evidence shows that miRNAs expression is deregulated in both solid and hematological malignancies [10, 11] and that deregulated miRNAs can induce and/or maintain a leukemogenic state.

In this study, we performed miRNA expression profiling (miEP) of Lin-CD34+CD38− and Lin-CD34-CD38- cells isolated from 5 CML patients and 4 healthy donors. This analysis identified a set of miRNAs aberrantly expressed in CML LSCs. In order to identify those miRNAs involved in the LSC-specific TKI escape, miRNAs whose expression is deregulated in CML independently from BCR-ABL kinase activity were selected. Our analysis allowed us to identify three novel miRNA/mRNA networks that confer BCR-ABL-independent TKI resistance to CML LSCs.

## RESULTS

### miRNA expression profiling of CML Lin-CD34-CD38- and Lin-CD34+CD38− cells

**Figure 1:**
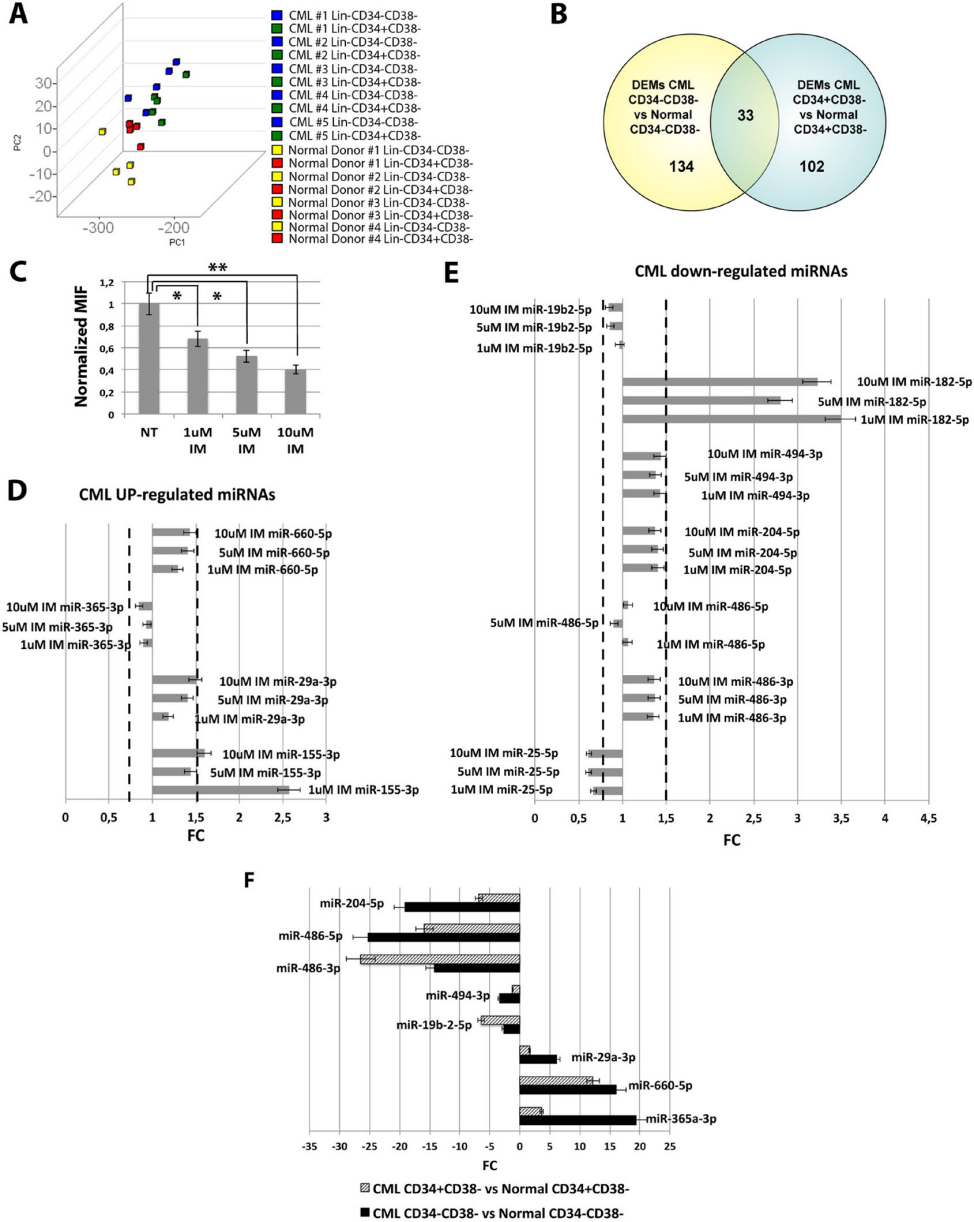
microRNAs expression data analysis (**A**) The Principal Component Analysis (PCA) graph of global miRNA expression data was computed using GenEx software version 6; CML Lin-CD34-CD38- are shown as blue cubes; CML Lin-CD34+CD38− are shown as green cubes, Normal Donor Lin-CD34-CD38- are shown as yellow cubes; CML Lin-CD34+CD38− are shown as red cubes. (**B**) Venn diagram showing the intersection of the lists of differentially expressed miRNAs (DEMs) in the comparison CML Lin-CD34-CD38- vs Normal Donor Lin-CD34-CD38- and CML Lin-CD34+CD38- vs Normal Donor Lin-CD34+CD38-. (**C**) Normalized MIF of intracellular p-CrkL levels in K562 cells after IM treatment. Untreated control pCRKL MIF was set to 1 to compare K562 before and after treatment with IM. The results are expressed as mean values ±SEM (*n* = 3), *p < 0.05, **p < 0.01 in untreated versus IM-treated cells. p-value from one-way ANOVA is 0.00002 (**D**) Real-Time PCR results showing expression levels of CML up-regulated miRNAs in K562 cells after IM treatment. (**E**) Real-Time PCR results showing expression levels of CML down-regulated miRNAs in K562 cells after IM treatment. Data are presented as Fold Change (FC) ± SEM (*n* = 3). FC of the untreated control was set to 1 to compare K562 before and after treatment with IM. BCR-ABL- independent miRNAs were identified as miRNAs with FC < 1.5 and > 0.67. (**F**) Relative expression level expressed as Fold Change (FC) of selected BCR-ABL-independent miRNAs in the comparisons CML Lin-CD34-CD38- vs Normal Donor Lin-CD34-CD38- (black bars) and CML Lin-CD34+CD38- vs Normal Donor Lin-CD34+CD38- (striped bars). Abbreviations: MIF indicates Mean Fluorescence Intensity; IM, Imatinib Mesylate; NT, Not Treated.

In order to shed light on the molecular properties of the CML stem cell compartment, we performed miEP on Lin-CD34-CD38- and Lin-CD34+CD38− cells from 5 CML patients and 4 healthy donors. To explore the relationships between samples, we performed a Principal Component Analysis (PCA). Figure 1A shows that the CML samples clustered together and were clearly separated from control samples. Of note, PCA revealed that CML Lin-CD34-CD38- are closer to leukemic CD34+CD38+ and normal CD34+ subfractions whereas their normal counterparts cluster separately, in agreement with our previous findings on the gene expression profile [6]. Next, differentially expressed miRNAs (DEMs) in the comparison CML vs normal donors for each cell population were identified by two-tail unpaired *t*-Student test as described in Materials and Methods (Supplementary Table 2 and Supplementary Table 3). This analysis identified 134 DEMs in the comparison CML Lin-CD34-CD38-vs Normal Donor Lin-CD34-CD38− and 102 DEMs in the comparison CML Lin-CD34+CD38−vs Normal Donor Lin-CD34+CD38−. 33 miRNAs resulted concordantly deregulated in both cell populations (Figure 1B, Table 1, Supplementary Tables 5 and 6).

**Table 1:** Common deregulated miRNAs in the comparison CML LSCs vs normal HSCs

miRNA ID	FC CML Lin-CD34-CD38- vs Normal Lin-CD34-CD38-	*P*-Value CML Lin-CD34-CD38- vs Normal Lin-CD34-CD38-	FC CML Lin-CD34+CD38− vs Normal Lin-CD34+CD38−	*P*-Value CML Lin-CD34+CD38−vs Normal Lin-CD34+CD38−
hsa-miR-193b-3p	65,71	0,0007	61,54	0,0027
hsa-miR-501-3p	34,23	0,0083	3,61	0,0199
hsa-miR-1207-5p	31,88	0,0001	8,58	0,0091
hsa-miR-188-5p	26,71	0,0078	14,38	0,0054
hsa-miR-708-5p	26,53	0,0128	74,18	0,0017
hsa-miR-362-3p	22,61	0,0009	11,59	0,0006
hsa-miR-365a-3p	19,35	0,0258	3,61	0,0306
hsa-miR-532-3p	17,63	0,0001	4,40	0,0073
hsa-miR-660-5p	16,08	0,0002	12,19	0,0005
hsa-miR-502-3p	13,13	0,0011	4,54	0,0056
hsa-miR-532-5p	12,63	0,0008	4,56	0,0028
hsa-miR-150-5p	12,22	0,0274	5,58	0,0033
hsa-miR-500a-5p	11,46	0,0052	4,76	0,0271
hsa-miR-362-5p	8,90	0,0011	4,80	0,0047
hsa-miR-29a-3p	6,12	0,0001	2,59	0,0044
hsa-miR-22-3p	3,54	0,0320	7,52	0,0003
hsa-miR-142-3p	3,53	0,0272	5,15	0,0037
hsa-miR-21-5p	3,08	0,0477	9,70	0,0007
hsa-miR-125b-5p	2,53	0,0315	−3,49	0,0428
hsa-miR-155-5p	2,36	0,0244	−4,19	0,0077
hsa-miR-140-5p	2,17	0,0347	3,10	0,0060
hsa-miR-875-3p	−2,35	0,0241	−4,62	0,0284
hsa-miR-494	−2,63	0,0197	−6,47	0,0509
hsa-miR-593-5p	−2,67	0,0178	−4,91	0,0106
hsa-miR-518d-3p	−2,72	0,0236	−7,90	0,0251
hsa-miR-609	−2,80	0,0270	−10,28	0,0303
hsa-miR-605	−3,86	0,0085	−10,94	0,0082
hsa-miR-134	−4,40	0,0009	−3,15	0,0375
hsa-miR-490-5p	−6,20	0,0377	−13,82	0,0044
hsa-miR-33b-3p	−7,33	0,0304	−7,71	0,0254
hsa-miR-625-3p	−14,05	0,0245	−3,05	0,0110
hsa-miR-486-3p	−14,21	0,0025	−26,46	0,0002
hsa-miR-204-5p	−19,09	0,0007	−6,84	0,0245
hsa-miR-486-5p	−25,31	0,0000	−15,86	0,0061

### Identification of BCR-ABL-independent deregulated miRNAs

miRNAs can be deregulated in CML through either a BCR-ABL kinase-dependent or a kinase-independent mechanism. To identify those miRNAs that were aberrantly expressed in CML LSCs through a BCR-ABL-independent mechanism, we selected miRNAs whose expression did not change after IM treatment. To this end, the BCR-ABL-positive K562 cells were treated with increasing doses of IM and the expression level of selected miRNAs was measured. For this analysis, 11 miRNAs, among the common 33 DEMs identified above, were selected based on their Fold Change (FC) in the comparison LSCs vs HSCs, and on their biological significance, evaluated as biological function, and expression level in other Myeloproliferative Neoplasms from our database [12]. The effectiveness of IM treatment on BCR-ABL kinase activity was assessed by measuring CrkL phosphorylation (p-CrkL) by flow cytometry. As shown in Figure 1C, the intracellular p-CrkL levels were significantly decreased in IM-treated samples compared to the untreated control. 8 miRNAs (miR-660-5p, miR-365-3p, miR-29a-3p, miR-19b2-3p, miR-494-3p, miR-204-5p, miR-486-5p and miR-486-3p) showed no statistically significant difference in their expression level after IM treatment (Figure 1D, 1E). These miRNAs were therefore classified as deregulated in CML with a BCR-ABL independent mechanism (Figure 1F).

### miR-29a-3p protects CML cells from TKIs-induced apoptosis

**Figure 2:**
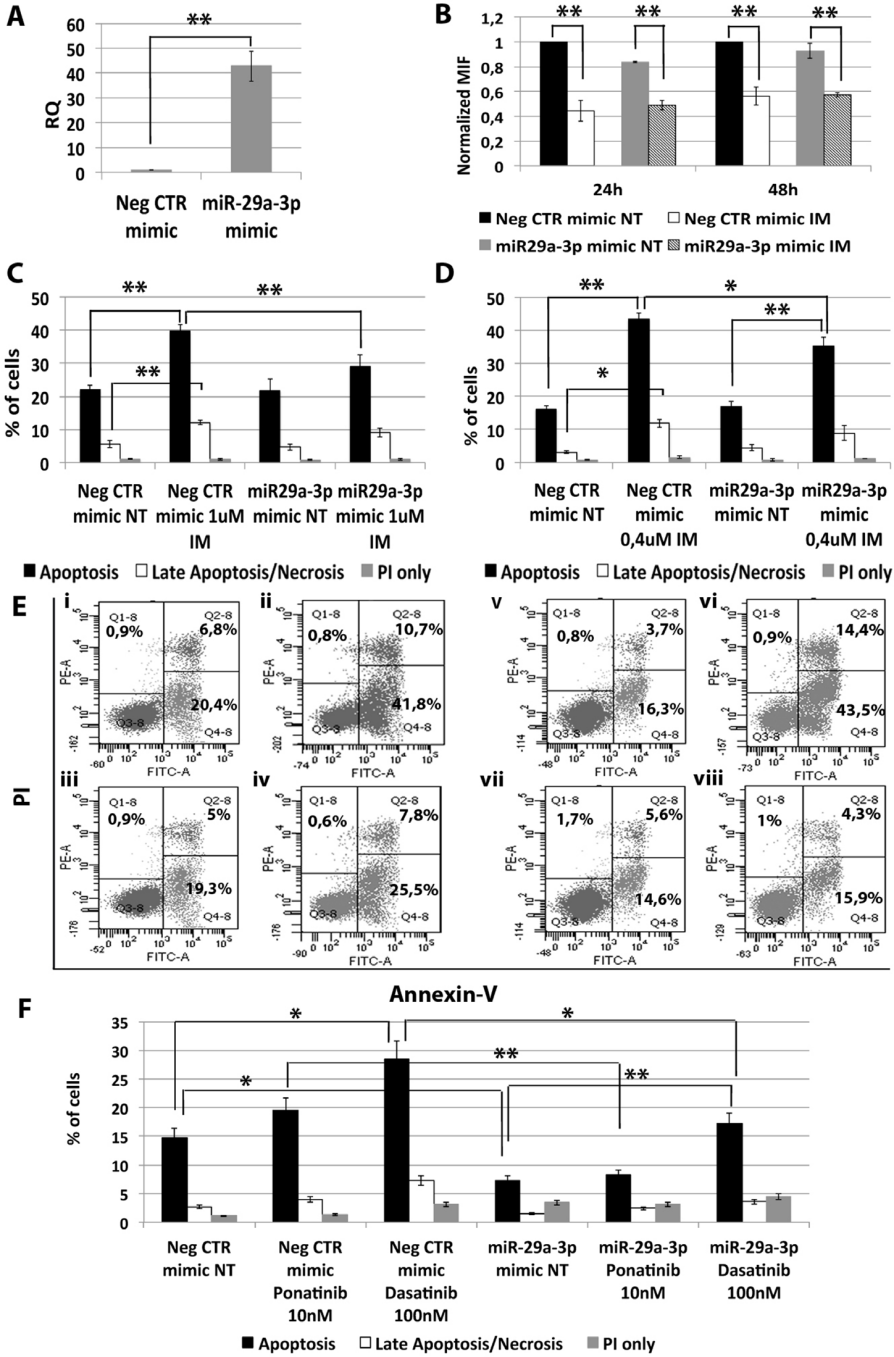
Effects of miR-29a-3p overexpression on K562 cells' response to TKIs (**A**) Expression levels of miR-29a-3p 24 hours after the last nucleofection as evaluated by qRT-PCR. Data are reported as RQ mean ± S.E.M of 3 independent experiments. (**B**) Normalized MIF of intracellular p-CrkL levels in K562 cells after miR-29a-3p overexpression. pCRKL MIF of the Neg CTR mimic untreated control was set to 1 to compare K562 before and after treatment with IM. The results are expressed as mean values ± SEM (*n* = 3). Results of Annexin V/PI staining on K562 cells after 24 h (**C**) and 48 h (**D**) of IM treatment (mean ± SEM; *n* = 3) *p < 0.05, **p < 0.01. Apoptotic cells (black bars in the histogram plot) are Annexin V bright and PI low, late apoptotic cells or necrotic cells (white bars in the histogram plot) are Annexin V and PI bright, grey bars represent cells bright for PI only. Representative dot plots for flow cytometry detection of Annexin V and PI staining at 24 h and 48 h after treatment are shown (**E**) i: Neg CTR mimic NT, ii: Neg CTR mimic IM 1 μM, iii: miR-29a-3p mimic NT, iv: miR-29a-3p mimic IM 1 μM, v: Neg CTR mimic NT, vi: Neg CTR mimic IM 0.4 μM, vii: miR-29a-3p mimic NT, viii: miR-29a-3p mimic IM 0.4 μM). (**F**) Results of Annexin V/PI staining on K562 cells after 24 h of 10 nM Ponatinib or 100 nM Dasatinib treatment (mean ± SEM; *n* = 3) *p < 0.05, **p < 0.01. Abbreviations: RQ, Relative Quantity; MIF indicates Mean Fluorescence Intensity; IM, Imatinib Mesylate; NT, Not Treated; 24 h, 24 hours; 48 h, 48 hours; PI, Propidium Iodide.

In order to assess whether miR-29a-3p up-regulation observed in CML LSCs is involved in the escape from TKIs treatment, miR-29a-3p was overexpressed in K562 cells and its effects on BCR-ABL kinase activity and cell survival in presence of TKIs treatment (1 μM IM for 24 h or 0.4 μM IM for 48 h, or 10 nM Ponatinib for 24 h, or 100 nM Dasatinib for 24 h) were evaluated. A significant overexpression of miR-29a-3p was detected by qRT-PCR 24 hours after last nucleofection (RQ ± SEM, 42.75 ± 6.04, *p* < 0.01) (Figure 2A). Analysis of p-CRKL levels showed that IM treatment significantly inhibited BCR-ABL kinase activity in all samples tested, regardless of miR-29a-3p overexpression (Figure 2B). Thus, miR-29a-3p does not directly affect BCR-ABL activity.

Interestingly, flow cytometric analysis of apoptosis performed by PI/Annexin V staining showed a significant decrease in the percentage of apoptotic cells in the sample transfected with miR-29a-3p mimic compared to the Neg CTR mimic, when incubated with IM (Figure 2C–2E) as well as with second and third generation TKIs (Figure 2F, Supplementary Figure 3).

Altogether, these data suggest that miR-29a-3p overexpression is able to protect K562 cells from TKIs-induced apoptosis without affecting BCR-ABL kinase activity.

### miR-29a-3p exerts its effects through *TET2* targeting

**Figure 3:**
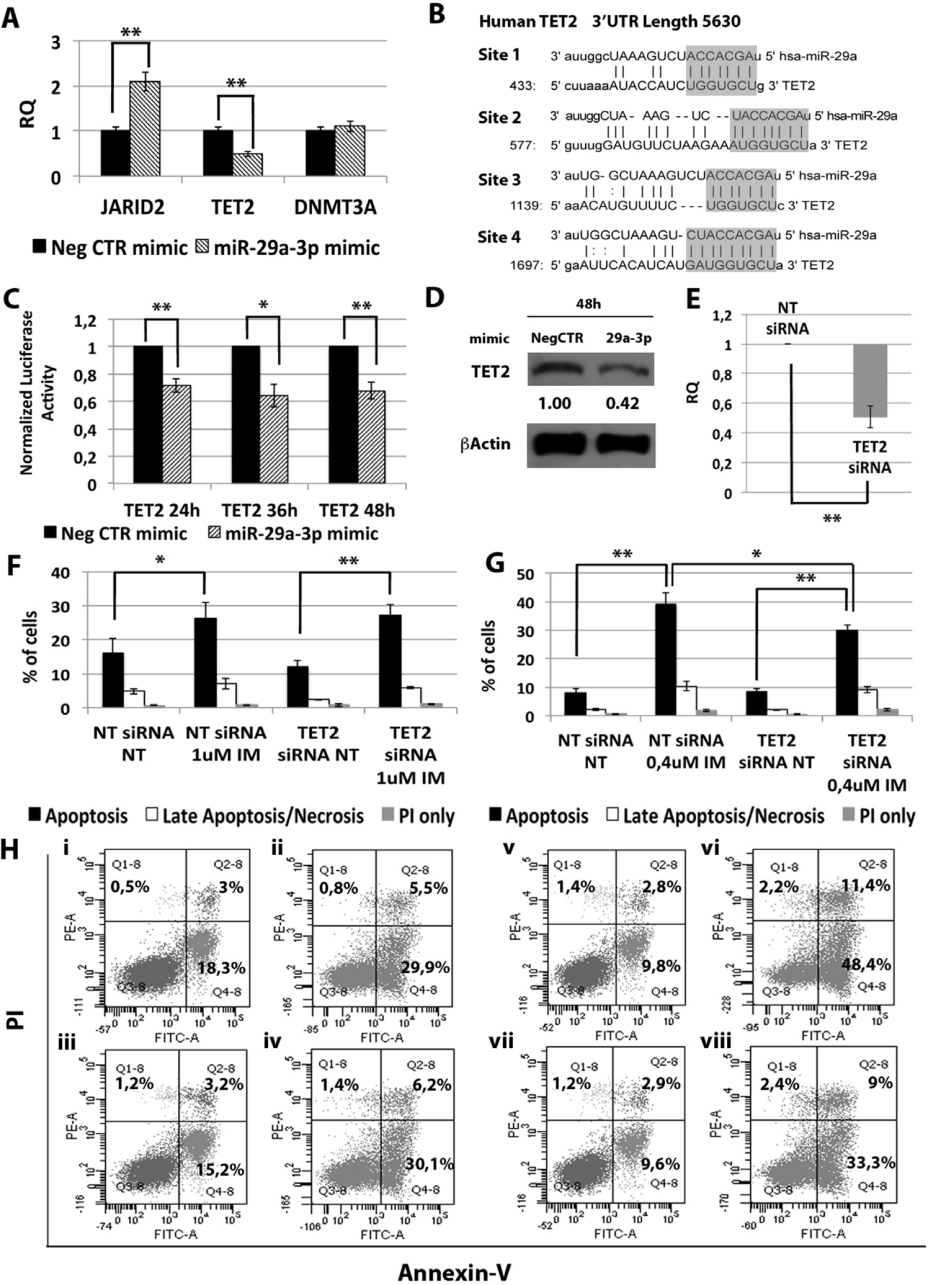
Effects of *TET2* silencing on K562 cells' response to IM (**A**) qRT-PCR analysis of miR-29a-3p predicted targets performed 24 h after miR-29a-3p transfection. Results were normalized to the NegCTR mimic sample (*n* = 3). (**B**) Representation of the four miR-29a-3p predicted binding sites in *TET2* 3′UTR sequence as reported by TargetScanHuman v7.0. The seed region of the miRNA is highlighted. (**C**) Normalized luciferase activity of K562 cells co-nucleofected with miR-29a-3p miRNA mimic and *TET2* 3′UTR reporter vector. Each bar represents the luciferase activity upon miRNA overexpression normalized on the value of the same 3′UTR luciferase vector upon Neg-mimic transfection (set to 1). Values are reported as mean ± SEM (*n* = 3). (**D**) Western blot analysis of TET2 protein levels in whole cell lysates from K562 cells overexpressing miR-29a-3p 48 hours after mimic nucleofection. TET2 protein level in miR-29a-3p overexpressing cells was compared with control sample nucleofected with mimic Negative Control (Neg CTR). β-actin was included as loading control. (**E**) TET2 mRNA expression levels 24 hours after the last nucleofection as evaluated by qRT-PCR. Data are reported as RQ mean ± S.E.M of 3 independent experiments. Results of Annexin V/PI staining on K562 cells after 24 h (**F**) and 48 h (**G**) of IM treatment (mean ± SEM; *n* = 3) *p < 0.05, **p < 0.01. Representative dot plots for flow cytometry detection of Annexin V and PI staining at 24 h and 48 h after treatment are shown (**H**), i: NT siRNA, ii: NT siRNA IM 1 μM, iii: TET2 siRNA NT, iv: TET2 siRNA IM 1 μM, v: NT siRNA NT, vi: NT siRNA IM 0.4 μM, vii: TET2 siRNA NT, viii: TET2 siRNA IM 0.4 μM. Abbreviations: RQ, Relative Quantity; NT siRNA, Non-targeting siRNA; siRNA, small interfering RNA; IM, Imatinib Mesylate; NT, Not Treated; 24 h, 24 hours; 36 h, 36 hours; 48 h, 48 hours; PI, Propidium Iodide.

In order to unravel the molecular mechanisms underlying the effects of miR-29a-3p on K562 cells, we investigated the mRNA expression level of miR-29a-3p putative targets identified through the TargetScan database (Figure 3B) (http://targetscan.org release 7.0). qRT-PCR was performed upon miR-29a-3p overexpression and the relative quantity of putative miR-29a-3p targets (i.e. *JARID2*, *TET2* and *DNMT3A*) was calculated. As shown in Figure 3A, only *TET2* (tet methylcytosine dioxygenase 2) mRNA was downregulated upon miR-29a-3p overexpression (RQ ± SEM, 0.49 ± 0.08, *p* < 0.01). To validate the direct interaction between miR-29a-3p and TET2, 3′UTR luciferase reporter assay was performed. The overexpression of miR-29a-3p did not affect the luciferase activity for the sample transfected with no 3′UTR, whilst it induced a statistically significant reduction in luciferase activity in the sample transfected with *TET2* 3′UTR reporter construct (Figure 3C), thus demonstrating for the first time that miR-29a-3p targets *TET2* 3′UTR. Furthermore, western blot (WB) analysis confirmed TET2 protein downregulation after miR-29a-3p overexpression (Figure 3D).

Next, we investigated whether *TET2* downregulation was able to reproduce the effects on IM sensitivity observed upon miR-29a-3p overexpression. To this end, *TET2* expression was silenced in K562 cells by means of *TET2* siRNAs. qRT-PCR analysis confirmed the downregulation of *TET2* mRNA in TET2 siRNA sample compared to the control (RQ ± SEM, 0.507 ± 0.07, *p* < 0.01) (Figure 3E). Twenty-four hours after last nucleofection, K562 cells were treated with either 1 μM IM for 24 h or 0.4 μM IM for 48 h, and p-CRKL intracellular levels and apoptosis were evaluated. In agreement with the results obtained upon miR-29a-3p overexpression, *TET2* silencing did not affect IM inhibition on BCR-ABL kinase activity (Supplementary Figure 2A). Moreover, our data showed a statistically significant decrease in the percentage of apoptotic cells in TET2 siRNA sample compared to the NTsiRNA (Figure 3F–3H). Altogether, these results demonstrate that TET2 silencing is able to protect cells from IM-induced apoptosis, thus phenocopying the effects mediated by miR-29a-3p overexpression.

### miR-660-5p protects CML cells from TKIs-induced apoptosis

**Figure 4:**
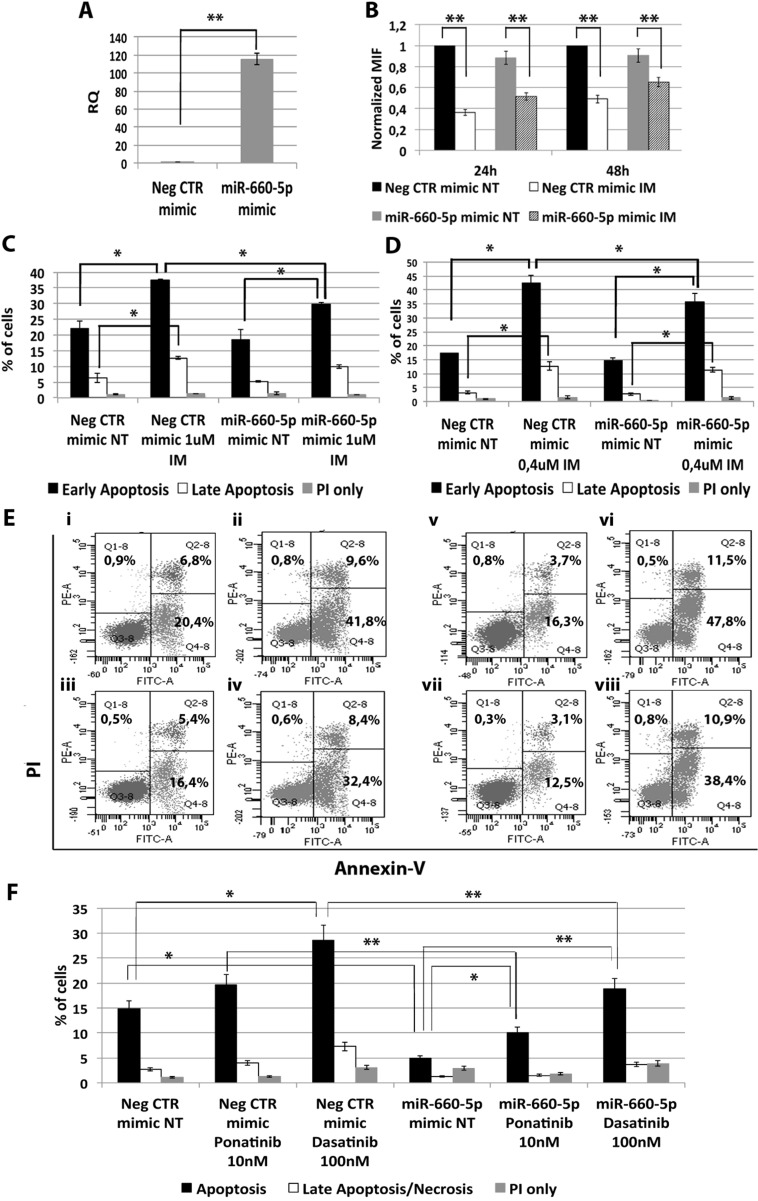
Effects of miR-660-5p overexpression on K562 cells' response to TKIs (**A**) Expression levels of miR-660-5p 24 hours after the last nucleofection as evaluated by qRT-PCR. Data are reported as RQ mean ± S.E.M of 3 independent experiments. The results are expressed as mean values ± SEM (**B**) Normalized MIF of intracellular p-CrkL levels in K562 cells after miR-660-5p overexpression. pCRKL MIF of the Neg CTR mimic untreated control was set to 1 to compare K562 before and after treatment with IM. The results are expressed as mean values ± SEM (*n* = 3) *p < 0.05, **p < 0.01. Results of Annexin V/PI staining on K562 cells after 24 h (**C**) and 48 h (**D**) of IM treatment (mean ± SEM; *n* = 3). Representative dot plots for flow cytometry detection of Annexin V and PI staining at 24 h and 48 h after treatment are shown (**E**) i: Neg CTR mimic NT, ii: Neg CTR mimic IM 1 μM, iii: miR-660-5p mimic NT, iv: miR-660-5p mimic IM 1 μM, v: Neg CTR mimic NT, vi: Neg CTR mimic IM 0,4 μM, vii: miR-660-5p mimic NT, viii: miR-660-5p mimic IM 0,4 μM). (**F**) Results of Annexin V/PI staining on K562 cells after 24h of 10nM Ponatinib or 100nM Dasatinib treatment (mean ± SEM; *n* = 3) *p < 0.05, **p < 0.01. Abbreviations: RQ, Relative Quantity; MIF indicates mean fluorescence intensity; IM, imatinib mesylate; NT, Not Treated; 24 h, 24 hours; 48 h, 48 hours; PI, Propidium Iodide.

Among miRNAs up-regulated in CML LSCs with a BCR-ABL-independent mechanism, our analysis pointed out miR-660-5p. In order to test whether its up-regulation is able to affect TKI sensitivity, K562 cells were transfected with either miR-660-5p mimic or Neg CTR mimic and BCR-ABL kinase activity and cell survival were evaluated by flow cytometric analysis. A significant overexpression of miR-660-5p was detected by qRT-PCR (RQ ± SEM, 115.29 ± 6.28, *p* < 0.01) (Figure 4A). Flow cytometric analysis of p-CRKL intracellular level showed no statistically significant difference between cells transfected with Neg CTR mimic and miR-660-5p mimic (Figure 4B). Moreover, our data showed a statistically significant decrease in the percentage of apoptotic cells in the miR-660-5p mimic sample compared to the Neg CTR mimic after TKIs treatment (Figure 4C–4F, Supplementary Figure 3). These data demonstrate that miR-660-5p overexpression is able to protect K562 cells from TKIs induced apoptosis without affecting BCR-ABL kinase activity.

**Figure 5:**
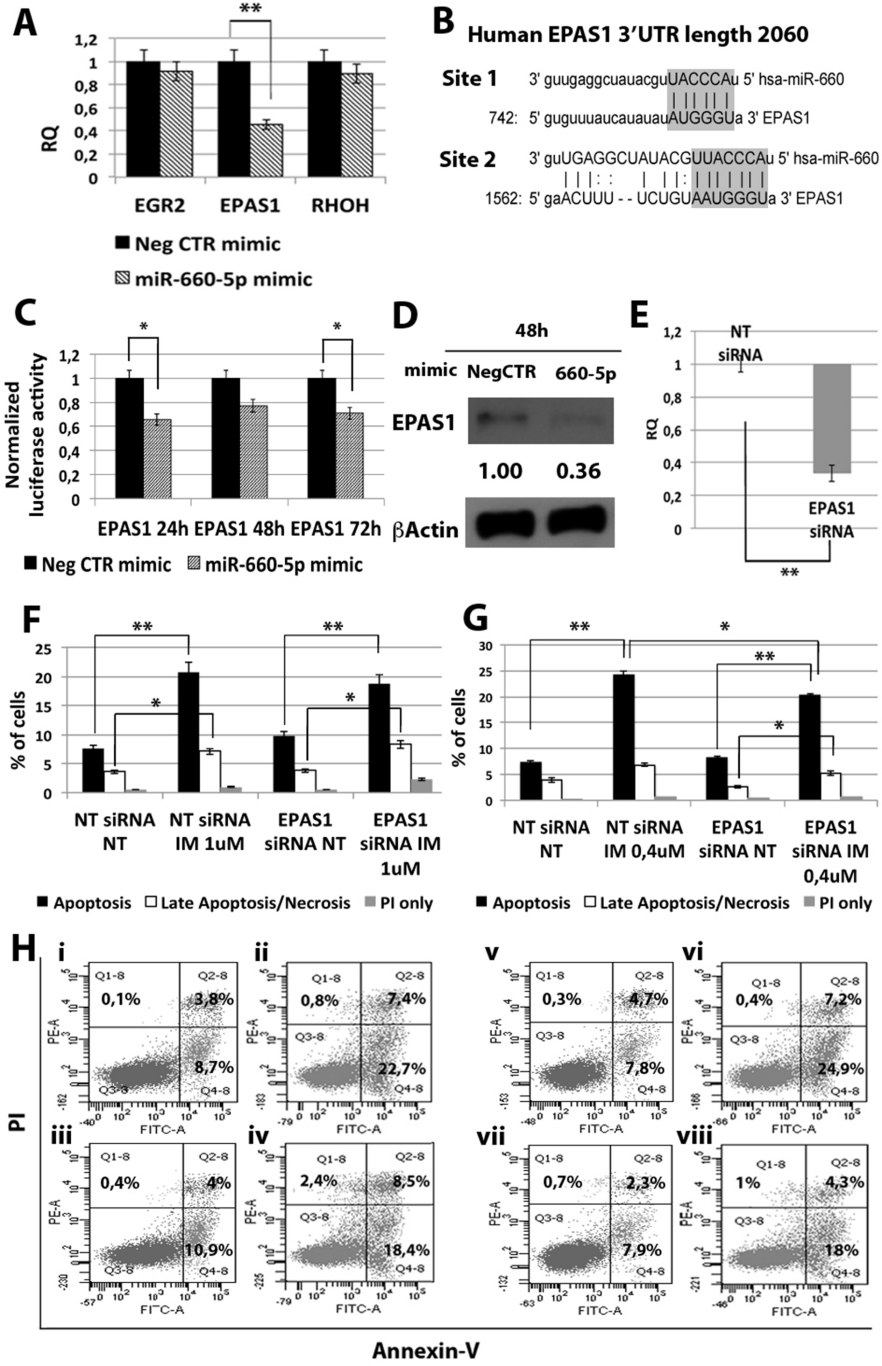
Effects of *EPAS1* silencing on K562 cells' response to IM (**A**) qRT-PCR analysis of miR-660-5p predicted targets performed 24 h after miR-660-5p transfection. Results were normalized to the NegCTR mimic sample (*n* = 3). (**B**) Representation of the two miR-660-5p predicted binding sites in *EPAS1* 3′UTR sequence as reported by TargetScanHuman v7.0. The seed region of the miRNA is highlighted. (**C**) Normalized luciferase activity of K562 cells co-nucleofected with miR-660-5p miRNA mimic and *EPAS1* 3′UTR reporter vector. Each bar represents the luciferase activity upon miRNA overexpression normalized on the value of the same 3′UTR luciferase vector upon Neg-mimic transfection (set to 1). Values are reported as mean ± SEM (*n* = 3) *p < 0.05, **p < 0.01. (**D**) Western blot analysis of EPAS1 protein levels in whole cell lysates from K562 cells overexpressing miR-660-5p 48 hours after mimic nucleofection. EPAS1 protein level in miR-660-5p overexpressing cells was compared with control sample nucleofected with mimic Negative Control (Neg CTR). β-actin was included as loading control. (**E**) *EPAS1* mRNA expression levels 24 hours after the last nucleofection as evaluated by qRT-PCR. Data are reported as RQ mean ± S.E.M of 3 independent experiments. Results of Annexin V/PI staining on K562 cells transfected with EPAS1 siRNA after 24 h (**F**) and 48 h (**G**) of IM treatment (mean ± SEM; *n* = 3). Representative dot plots for flow cytometry detection of Annexin V and PI staining at 24 h and 48 h after treatment are shown (**H**), i: NT siRNA NT, ii: NT siRNA IM 1 μM, iii: EPAS1 siRNA NT, vi: EPAS1 siRNA IM 1 μM, v: NT siRNA NT, vi: NT siRNA IM 0.4 μM, vii: EPAS1 siRNA NT, viii: EPAS1 siRNA IM 0.4 μM. *p < 0.05, **p < 0.01. Abbreviations: RQ, Relative Quantity; MIF indicates Mean Fluorescence Intensity; IM, Imatinib Mesylate; NT, Not Treated; 24 h, 24 hours; 48 h, 48 hours; PI, Propidium Iodide.

Then, in order to shed light on the molecular mechanisms responsible for miR-660-5p effects, we searched on the TargetScan database for putative miR-660-5p targets. Between all predicted targets tested, only *EPAS1* (endothelial PAS domain protein 1) transcript showed a statistically significant downregulation upon miR-660-5p overexpression (RQ ± SEM, 0.45 ± 0.07, *p* < 0.01) (Figure 5A). As shown in Figure 5B, EPAS1 3′ untraslated region (3′UTR) displays two putative miRNA recognition elements (MRE), as reported by the target prediction tools TargetScanHuman andmicroRNA.org (www.microrna.org). To validate the direct interaction between miR-660-5p and EPAS1, 3′UTR luciferase reporter assay was performed: results showed a statistically significant reduction in normalized luciferase activity in K562 cells co-nucleofected with miR-660-5p miRNA mimic and EPAS1 reporter construct (Figure 5C), thus demonstrating for the first time that miR-660-5p targets EPAS1 3′UTR. Furthermore, western blot (WB) analysis confirmed EPAS1 protein downregulation after miR-660-5p overexpression (Figure 5D).

Next, EPAS1 expression was silenced in K562 cells by means of transfection with *EPAS1* siRNA (EPAS1 siRNA). qRT-PCR analysis confirmed the downregulation of *EPAS1* mRNA in EPAS1 siRNA sample compared to the NTsiRNA (RQ ± SEM, 0.337 ± 0.05, *p* < 0.01) (Figure 5E). Twenty-four hours after last transfection, K562 cells were treated with either 1uM IM for 24 h or 0.4 uM IM for 48h and cells were analyzed for apoptosis and pCRKL levels. Our results showed that *EPAS1* silencing does not affect IM inhibitory effect on BCR-ABL kinase activity (Supplementary Figure 2B). On the other hand, *EPAS1* silencing was able to protect K562 cells from IM-induced apoptosis (Figure 5F–5H). Thus, these data demonstrate that *EPAS1* silencing is able to reproduce the effects exerted by miR-660-5p overexpression, suggesting that miR-660-5p is able to protect cells from IM-induced apoptosis through *EPAS1* targeting.

### miR-494-3p sensitizes CML cells to TKIs-induced apoptosis

**Figure 6:**
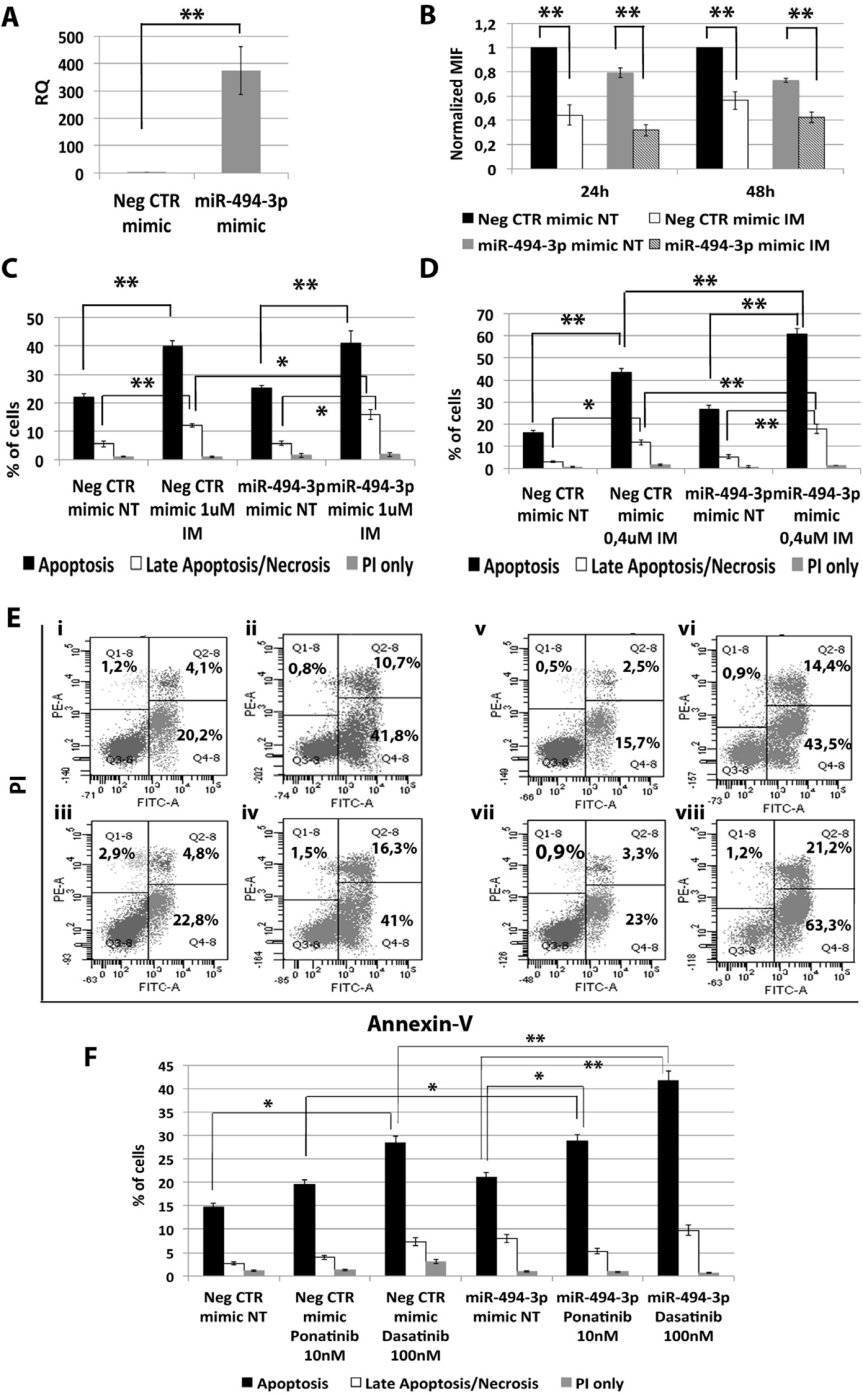
Effects of miR-494-3p overexpression on K562 cells' response to TKIs (**A**) Expression levels of miR-494-3p 24 hours after the last nucleofection as evaluated by qRT-PCR. Data are reported as RQ mean ± S.E.M of 3 independent experiments. (**B**) Normalized MIF of intracellular p-CrkL levels in K562 cells after miR-494-3p overexpression. pCRKL MIF of the Neg CTR mimic untreated control was set to 1 to compare K562 before and after treatment with IM. The results are expressed as mean values ± SEM (*n* = 3). Results of Annexin V/PI staining on K562 cells after 24 h (**C**) and 48 h (**D**) of IM treatment (mean ± SEM; *n* = 3) *p < 0.05, **p < 0.01. Representative dot plots for flow cytometry detection of Annexin V and PI staining at 24 h and 48 h after treatment are shown (**E**) i: Neg CTR mimic NT, ii: Neg CTR mimic IM 1μM, iii: miR-494-3p mimic NT, iv: miR-494-3p mimic IM 1 μM, v: Neg CTR mimic NT, vi: Neg CTR mimic IM 0.4 μM, vi: miR-494-3p mimic NT, viii: miR-494-3p mimic IM 0.4 μM). (**F**) Results of Annexin V/PI staining on K562 cells after 24 h of 10 nM Ponatinib or 100 nM Dasatinib treatment (mean ± SEM; *n* = 3) *p < 0.05, **p < 0.01. Abbreviations: RQ, Relative Quantity; MIF indicates mean fluorescence intensity; IM, imatinib mesylate; NT, Not Treated; 24 h, 24 hours; 48 h, 48 hours; PI, Propidium Iodide.

Among miRNAs down-regulated in CML LSCs with a BCR-ABL-independent mechanism, our analysis highlighted miR-494-3p. To assess whether the down-regulation of miR-494-3p observed in CML LSCs can control response to first, second and third generation of TKIs, we overexpressed miR-494-3p in K562 cells and evaluated apoptosis and p-CRKL levels after TKIs treatment. qRT-PCR showed a significant up-regulation of miR-494-3p after miR-494-3p mimic transfection (RQ ± SEM, 372.89 ± 86.92, *p* < 0.01) (Figure 6A). Flow cytometric analysis of p-CRKL intracellular level showed no statistically significant difference between cells transfected with Neg CTR mimic and miR-494-3p mimic (Figure 6B). These data suggest that miR-494-3p does not directly affect BCR-ABL kinase activity. Moreover, our data showed a statistically significant increase in the percentage of both apoptotic cells and late apoptotic/necrotic cells in miR-494-3p mimic sample compared to the Neg CTR mimic (Figure 6C–6F, Supplementary Figure 3). These results demonstrate that miR-494-3p overexpression is able to sensitize K562 cells to TKIs-induced apoptosis without affecting BCR-ABL kinase activity.

### miR-494-3p exerts its effects through *c-MYC* targeting

**Figure 7:**
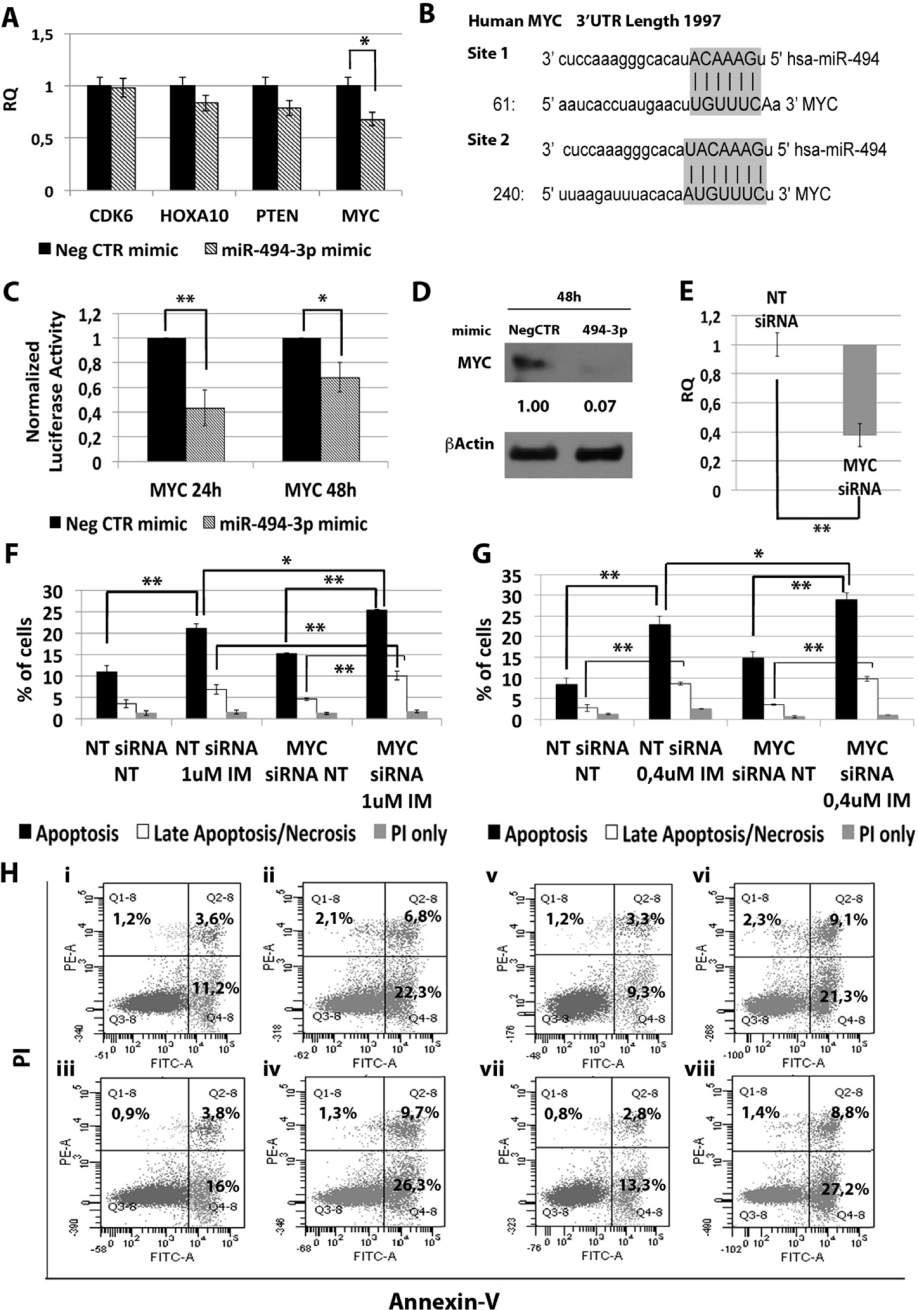
Effects of MYC silencing on K562 cells' response to IM (**A**) qRT-PCR analysis of miR-494-3p predicted targets performed 24h after miR-494-3p transfection. Results were normalized to the NegCTR mimic sample (*n* = 3). (**B**) Representation of the two miR-494-3p predicted binding sites in *MYC* 3′UTR sequence as reported by TargetScanHuman v7.0. The seed region of the miRNA is highlighted. (**C**) Normalized luciferase activity of K562 cells co-nucleofected with miR-494-3p miRNA mimic and *MYC* 3′UTR reporter vector. Each bar represents the luciferase activity upon miRNA overexpression normalized on the value of the same 3′UTR luciferase vector upon Neg-mimic transfection (set to 1). Values are reported as mean ± SEM (*n* = 3). (**D**) Western blot analysis of MYC protein levels in whole cell lysates from K562 cells overexpressing miR-494-3p 48 hours after mimic nucleofection. MYC protein level in miR-494-3p overexpressing cells was compared with control sample nucleofected with mimic Negative Control (Neg CTR). β-actin was included as loading control. (**E**) *MYC* mRNA expression levels 24 hours after the last nucleofection as evaluated by qRT-PCR. Data are reported as RQ mean ± S.E.M of 3 independent experiments. Results of Annexin V/PI staining on K562 cells after 24 h (**F**) and 48 h (**G**) of IM treatment (mean ± SEM; *n* = 3) *p < 0.05, **p < 0.01. Representative dot plots for flow cytometry detection of Annexin V and PI staining at 24 h and 48 h after treatment are shown (**H**) i: NT siRNA NT, ii: NT siRNA IM 1 μM, iii: MYC siRNA NT, iv: MYC siRNA IM 1 μM, v: NT siRNA NT, vi: NT siRNA IM 0.4 μM, vii: MYC siRNA NT, viii: MYC siRNA IM 0.4 μM). Abbreviations: RQ, Relative Quantity; NT siRNA, Non-targeting siRNA; siRNA, small interfering RNA; IM, Imatinib Mesylate; NT, Not Treated; 24 h, 24 hours; 48 h, 48 hours; PI, Propidium Iodide.

In order to identify the molecular mechanism responsible for the effects of miR-494-3p on K562 cells sensitivity to TKIs, we performed a computational analysis for predicted miR-494-3p targets on TargetScan database (Figure 7B). Among all predicted targets tested (i.e. *CDK6*, *HOXA10*, *PTEN* and *c-MYC*), only *c-MYC* (v-myc avian myelocytomatosis viral oncogene homolog) transcript showed a statistically significant downregulation after miR-494-3p overexpression (RQ ± SEM, 0.68 ± 0.08, *p* < 0.01) (Figure 7A). To validate the direct interaction between miR-494-3p and *c-MYC* we performed 3′UTR luciferase reporter assay. Our results showed a statistically significant reduction in luciferase activity in K562 cells co-nucleofected with miR-494-3p miRNA mimic and *c-MYC* reporter construct compared to the sample transfected with the 3′UTR-less vector (Figure 7C), thus demonstrating a direct interaction between miR-494-3p and *c-MYC* 3′UTR. Moreover, western blot (WB) analysis confirmed c-MYC protein downregulation after miR-494-3p overexpression (Figure 7D).

Next, *c-MYC* expression was silenced in K562 cells by means of siRNAs targeting *c-MYC* (MYC siRNA). qRT-PCR analysis confirmed *c-MYC* downregulation in MYC siRNA sample compared to the control (RQ ± SEM, 0.38 ± 0.01, *p* < 0.01) (Figure 7E). In agreement with the results obtained upon miR-494-3p overexpression, *c-MYC* silencing did not affect IM inhibitory ability on BCR-ABL kinase activity (Supplementary Figure 2C). Moreover, our data showed a statistically significant increase in the percentage of both apoptotic and late apoptotic/necrotic cells in the MYC siRNA sample compared to the NTsiRNA (Figure 7F–7H).

### miR-29a-3p, miR-494-3p and miR-660-5p affect TKI sensitivity of CML LSCs

**Figure 8:**
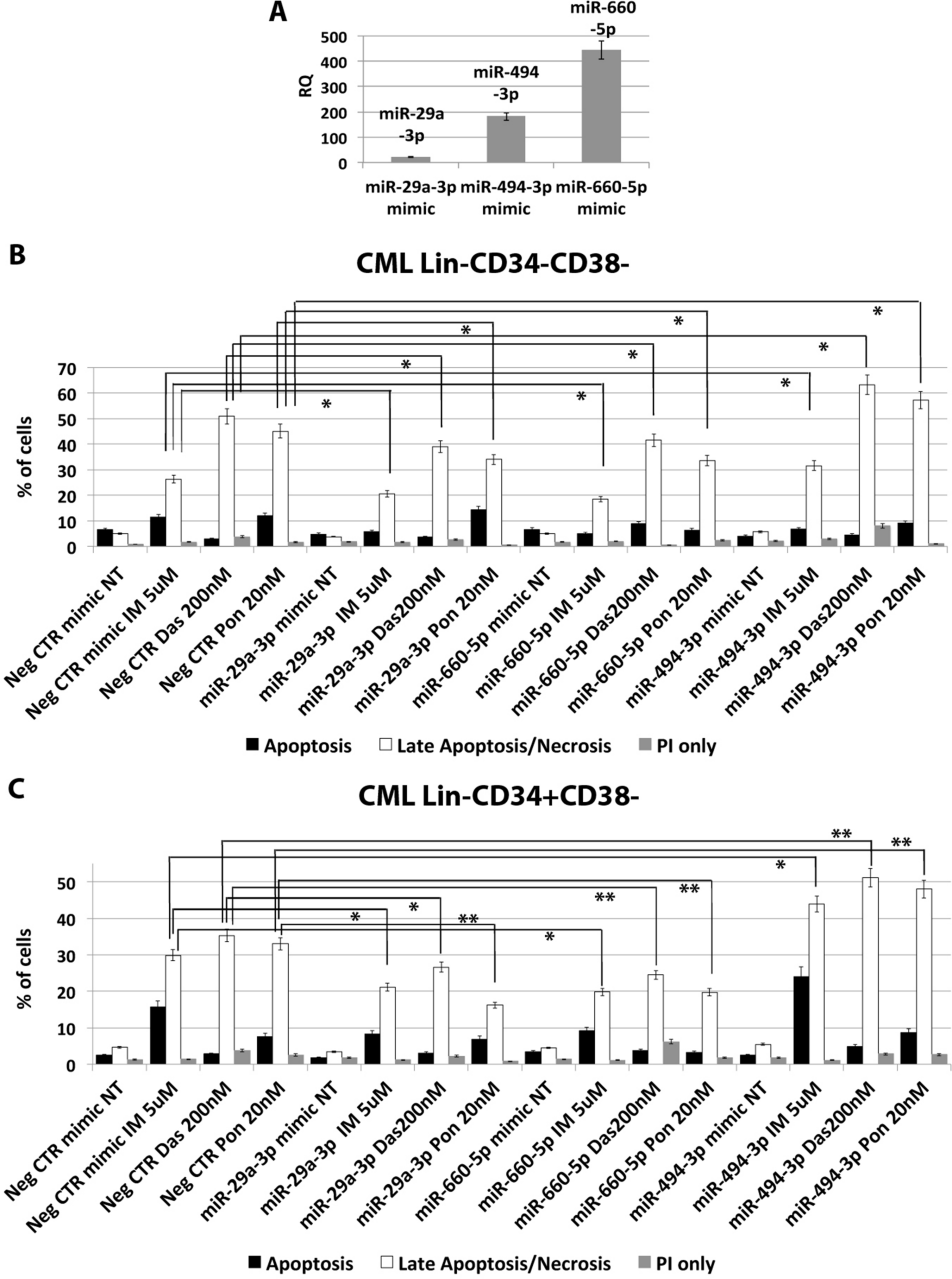
miR-29a-3p, miR-660-5p and miR494-3p overexpression in HSPCs isolated from CML patients (**A**) Expression levels of miR-29a-3p, miR-494-3p and miR-660-5p 24 hours after the last nucleofection as evaluated by qRT-PCR. Data are reported as RQ mean ± S.E.M of 3 independent experiments. Results of Annexin V/PI staining on Lin-CD34-CD38− (**B**) and Lin-CD34+CD38− (**C**) cells isolated from CML patients after 48 h of 5 μM IM, or 200 nM Dasatinib or 20 nM Ponatinib treatment (mean ± SEM; *n* = 3) *p < 0.05, **p < 0.01. Abbreviations: RQ, Relative Quantity; IM, imatinib mesylate; Das, Dasatinib; Pon, Ponatinib; NT, Not Treated; 48 h, 48 hours; PI, Propidium Iodide.

In order to assess whether the deregulated expression of miR-29a-3p, miR-494-3p and miR-660-5p is able to impact on CML LSCs' sensitivity to TKIs, we overexpressed these miRNAs in Lin- cells isolated from 5 CML patients and evaluated apoptosis after TKIs treatment (10 μM IM for 24 h or 5 μM IM for 48 h, or 20 nM Ponatinib for 48 h, or 200 nM Dasatinib for 48 h). A significant overexpression of miR-29a-3p, miR-494-3p and miR-660-5p was detected by qRT-PCR (RQ ± SEM, 20.6 ± 1.8, 181.4 ± 16.1, 443.8 ± 37.5, respectively *p* < 0.05) (Figure 8A). Interestingly, flow cytometric analysis of apoptosis performed by PI/Annexin V staining showed a significant decrease in the percentage of cells in late apoptosis/necrosis in the samples transfected with either miR-29a-3p mimic or miR-660-5p mimic compared to the Neg CTR mimic (Figure 8B, 8C). Moreover, our data showed a strong increase in the percentage of late apoptotic/necrotic cells in the sample overexpressing miR-494-3p compared to the control (Figure 8B, 8C, Supplementary Tables 8 and 9). These differences were detected on the whole hematopoietic progenitor cells population (data not shown), as well as on the more primitive cell fractions, Lin-CD34-CD38- and Lin-CD34+CD38− (Figure 8B, 8C).

## DISCUSSION

Treatment of CML has been notably improved in the past 15 years thanks to the development of TKIs blocking BCR-ABL kinase activity. However, the majority of patients achieving remission with imatinib continue to test positive for BCR-ABL transcript by RT-PCR. Indeed, in recent years, several *in vitro* evidence suggested that CML LSCs are intrinsically resistant to Imatinib. Chu *et al*. [5] demonstrated that BCR-ABL+ stem cells persist in the bone marrow of CML patients in prolonged remission on Imatinib treatment. Corbin *et al*. [4] demonstrated that CML LSCs survive Imatinib with a mechanism independent from BCR-ABL kinase activity. Notably, *in vivo*, only approximately 50% of patients who had stopped Imatinib therapy remained long-term disease free, at the molecular level, suggesting, in many cases, the persistence of LSCs [13]. Thus, treatment targeting BCR-ABL does not completely eliminate CML stem cells, and disease eradication will require additional therapeutic strategies. In contrast, Mustjoki et al. recently reported that *in vivo* TKI therapy is able to clear most of Ph+ cells from the stem cell compartment [13]. These differences might be due to the different time points applied for LSCs analysis, usually at diagnosis for *in vitro* studies, at later time points in the work by Mustjoki et al. or to technical limitations that might have prevented the identification of few residual LSCs. Typically, CML stem cells have been defined as lineage-negative CD34-positive CD38-negative (Lin-CD34+CD38−) cells. However, our group recently identified a new subset of LSCs negative for CD34 expression, therefore identified as Lin-CD34-CD38- cells [6]. Our data demonstrate that approximately one-third of Lin-CD34-CD38- cells are leukemic and capable of engraftment in immunodeficient mice. Moreover, these cells showed intrinsic resistance to IM both *in vitro* and *in vivo*. Therefore, Lin-CD34-CD38- CML cells can be seen as a stem cell subset insensitive to targeted therapy that may contribute to disease persistence.

In the last years, miRNAs expression has been reported to be deregulated in hematological malignancies [10], where it is responsible for disease development or maintenance.

Based on these premises, in order to gain further understanding on the molecular features of the CML stem cell compartment, we analyzed the miEP of both Lin-CD34-CD38- and Lin-CD34+CD38− LSCs, and miRNAs deregulated in both cell fractions were selected. Our analysis identified 33 differentially expressed miRNAs in the comparison LSCs vs HSCs (Figure 1B). As Corbin et al. demonstrated that LSCs escape from IM treatment independently from BCR-ABL kinase activity, we sought to identify those miRNAs that were deregulated in CML LSCs with a mechanism that is independent from BCR-ABL. To this aim, BCR-ABL-positive K562 cells were treated with increasing doses of IM and miRNAs, whose expression did not change after IM treatment, were selected and classified as BCR-ABL-independent miRNAs. This analysis unraveled 8 miRNAs, whose expression is deregulated in CML LSCs independently from BCR-ABL (Figure 1F). Next, in order to assess whether the deregulated expression of such miRNAs was able to affect TKIs-sensitivity, miRNA overexpression experiments were performed. As initial analysis, the function of selected miRNAs was studied in K562 cells, then, in order to validate our results, miRNA overexpression experiments were performed in Hematopoietic Stem/Progenitor Cells (HSPCs) isolated from CML patients collected in chronic phase before therapy. All eight BCR-ABL-independent miRNAs, except miR-486 whose role in CML has been recently described by Dr. Bhatia's group [14], were studied. Our results demonstrated that the overexpression of miR-204-5p, miR-19b-2-5p, and miR-365a-3p do not affect IM response in K562 cells (data not shown). On the other hand, miR-29a-3p, miR-660-5p and miR-494-3p showed a strong influence on TKIs sensitivity when overexpressed in BCR-ABL-positive cells. In particular, miR-29a-3p overexpression was able to protect cells from TKIs-induced apoptosis (Figure 2C–2F). Of note, IM-induced BCR-ABL kinase inhibition was not affected by miR-29a-3p expression level (Figure 2B), therefore demonstrating that miR-29a-3p up-regulation is able to confer IM-resistance with a BCR-ABL-independent mechanism. By means of 3′UTR luciferase reporter assay, we were able to demonstrate for the first time in the hematopoietic context the direct interaction between miR-29a-3p and *TET2* (Figure 3C). Moreover, *TET2* silencing mimicked the effects induced by miR-29a-3p overexpression, suggesting that miR-29a-3p protects cells from IM-induced apoptosis through *TET2* targeting. Han YC et al. provided evidence that miR-29a-3p may serve as oncogene during Acute Myeloid Leukemia (AML) development: ectopic expression of miR-29a induce the acquisition of self-renewal capacity by myeloid progenitors, biased myeloid differentiation, and the development of a myeloproliferative disorder [15]. Somatic loss of function mutations in *TET2* are frequently observed in myelodysplastic syndromes (MDS), myeloproliferative neoplasms (MPN), and AML [16, 17]. Recently, decreased *TET2* expression associated with t(4;6;11) rearrangement has been demonstrated to be involved in CML progression. Of note, the appearance of the *TET2* deletion was not associated with an increase of the BCR-ABL transcript level, suggesting a BCR-ABL-independent role for this gene in CML progression [18]. Our data strongly suggest that the up-regulation of miR-29a-3p observed in CML LSCs is responsible for *TET2* downregulation, which in turn is able to confer IM-resistance to CML LSCs. These findings imply that *TET2* could be involved in the pathogenesis of myeloid malignancies through deregulated gene expression in addition to inactivating mutations and provide a rationale for targeted therapies that impact *TET2* aberrant regulation.

Among miRNAs up-regulated in CML LSCs with a BCR-ABL-independent mechanism, our analysis pointed out miR-660-5p. miR-660-5p has been previously reported in the context of hematopoiesis as involved in polyploidization and megakaryocytes differentiation [19]. To our knowledge, this is the first report describing a role for miR-660-5p in myeloid malignancies. miR-660-5p overexpression was able to protect cells from TKIs-induced apoptosis (Figure 4C–4F). IM-induced BCR-ABL kinase inhibition was not affected by miR-660-5p expression level (Figure 4B), therefore demonstrating that miR-660-5p up-regulation is able to confer IM-resistance to K562 cells with a BCR-ABL-independent mechanism. Between miR-660-5p predicted targets, *EPAS1* showed significant downregulation upon miR-660-5p overexpression both at the mRNA and protein level (Figure 5A and 5D respectively). *EPAS1* silencing was able to protect cells from IM-induced apoptosis thus phenocopying the effects induced by miR-660-5p overexpression (Figure 5F–5H). *EPAS1*, also known as hypoxia-inducible factor 2 alpha (*HIF2A*), is a transcription factor critical for the adaptive response to hypoxia. *HIF2A* expression has been associated with aggressive disease phenotype, metastasis and resistance to therapy in several solid tumors, such as neuroblastoma [20], non-small cell lung cancer [21], and breast cancer [22]. On the other hand, recent evidence showed that in HIF-2a inhibits high-grade soft tissue sarcomas cell growth *in vivo* [23], suggesting a cell context-dependent role for *EPAS1* in tumor development. Our data strengthen this hypothesis, in fact, in the CML context, *EPAS1* down-regulation is involved in the escape from IM treatment of BCR-ABL-positve cells.

On the other hand, between miRNAs down-regulated in CML LSCs independently from BCR-ABL activity, our miEP data highlighted miR-494-3p (Figure 1E, 1F). miR-494 has been reported as an oncogenic miRNA in a variety of cancers including colorectal cancer [24], breast cancer [25], ovarian cancer [26], and prostate cancer [27]. However, in several solid tumors, i.e. oral and gastric cancer, miR-494-3p acts as an anti-oncogene by targeting *HOXA10* and *c-MYC* respectively [28, 29]. Our data demonstrated that miR-494-3p overexpression is able to sensitize K562 cells to TKIs-induced apoptosis without affecting BCR-ABL kinase acitvity (Figure 6C–6F). Thus suggesting that miR-494-3p acts as tumor suppressor miRNA in CML, where its downregulation is necessary for the intrinsic resistance to TKIs that characterizes LSCs. In order to identify the molecular mechanisms governing miR-494-3p-induced IM sensitization, 3′UTR luciferase assay was performed and *c-MYC* was identified as miR-494-3p target (Figure 7C). At the time this manuscript was in preparation, Tian C. et al. [30] reported *c-MYC* downregulation upon miR-494-3p overexpression in AML cell lines. To our knowledge, these are the first two reports demonstrating *c-MYC* targeting by miR-494-3p in hematological malignancies. Noteworthy, *c-MYC* silencing was able to sensitize cells to IM-induced apoptosis (Figure 7F–7H), thus mimicking the effects induced by miR-494-3p overexpression. Recently an interesting dual role for *c-MYC* in CML has been reported: Reavie L. *et al*. showed that both depletion and overexpression of *c-MYC* can severely impact CML progression, suggesting that a specific level of c-Myc protein is essential for CML induction and progression [31]. In this view, our results suggest that *c-MYC* overexpression induced by miR-494-3p downregulation in LSCs is strongly connected to BCR-ABL-independent TKI-resistance. This hypothesis opens the way for novel therapeutic strategies combining TKI and c-Myc inhibitors to effectively target CML LSCs.

Worth of note, our data also demonstrated that ectopic expression of miR-29a-3p, miR-494-3p and miR-660-5p was able to affect the response to first, second and third generation TKIs of HSPCs isolated from CML patients (Figure 8B, 8C), therefore suggesting that their deregulated expression observed in CML LSCs might be responsible for the escape from TKIs treatment *in vivo*.

In summary, our analysis uncovered several miRNAs aberrantly expressed in CML LSCs. In particular, we identified those miRNAs that are deregulated in LSCs independently from BCR-ABL kinase activity and therefore are likely to be involved in the BCR-ABL-independent resistance to TKI that characterizes CML LSCs. This work unraveled three mRNA/miRNA networks (miR-29a-3p/*TET2*, miR-660-5p/*EPAS1* and miR-494-3p/*c-MYC*) capable of affecting TKI-sensitivity in CML cells, thus leading to the design of novel targeted therapies aimed at eliminating TKI-resistant LSCs.

## MATERIALS AND METHODS

### Patients and samples

Leukemic cells were obtained from peripheral blood (PB) of 10 chronic phase Ph+ CML patients at diagnosis, 5 were analysed for miRNA expression profiling, 5 were used for miRNA overexpression experiments (Supplementary Table 1). Normal samples were leukapheresis products from 4 healthy donors receiving recombinant human granulocyte colony-stimulating factor (G-CSF; Lenograstim; Sanofi-Aventis). All subjects provided informed written consent, and the study was performed under the local Institutional Review Board's approved protocol (GIMEMA CML0811, clinicaltrials.gov identifier: 01535391; CE approval, Bologna, 13/09/2011, n. 70/2011/U). The study was conducted in accordance with the Declaration of Helsinki. Hematopoietic stem/progenitor cell purification and phenotypic analyses were performed as previously described [32, 33]. Aliquots of sorted Lin−CD34−CD38- and Lin−CD34+CD38- were reanalyzed by FACScan (Becton Dickinson) to assess their purities, which were 98.8% (± 0.5%) and 99.1% (± 0.8%), respectively (Supplementary Figure 1). As previously reported [6], FISH analysis and RQ-PCR for BCR-ABL transcript demonstrated that Ph+ cells were 70.5% (± 5%), and 32% (± 2%) of total Lin-CD34+CD38− and Lin-CD34-CD38- CML cells at diagnosis, respectively.

### RNA extraction

Total cellular RNA was harvested from 1 × 10^5^ cells from each sample using the miRNeasy Micro RNA isolation kit (QIAGEN), according to the manufacturer's instructions. RNA samples concentration and purity (assessed as 260/280 nm and 260/230 nm ratios) were evaluated by NanoDrop ND-1000 spectrophotometer (NanoDrop Technologies; Wilmington, DE), while RNA integrity was assessed by using the Agilent 2100 Bioanalyzer (Agilent Technologies; Waldbrunn, Germany).

### miRNA expression profiling

miEP was performed by using the miRCURY LNA^TM^ Universal RT microRNA PCR system, Ready-to-use Human miRNome Panels I and II (Exiqon, Copenhagen, Denmark). RT-qPCR data were analysed using the Exiqon GenEx software for the inter-plate calibration, the definition of a Ct=40 as detection cutoff, the labeling of not-amplified wells as missing data and the setting of a cutoff for miRNAs with a low call-rate (i.e. assays with a detection rate < 33,3%, i.e. assays not detected in 12 out of 18 samples, were excluded from analysis). For RT-qPCR data normalization, the comparative cycle threshold (CT) method was used by setting miR-23a-3p, miR-24-3p and miR-324-3p as reference miRNAs, as the best normalizers identified among the candidates by the NormFinder algorithm. The Relative Quantity (RQ) value was expressed as 2^−ΔΔCT^. Statistical analyses on ΔCT values between CML Lin-CD34-CD38- and Normal donor Lin-CD34-CD38− (set as calibrator) and between CML Lin-CD34+CD38− and Normal donor Lin-CD34+CD38- (set as calibrator) were performed by a two-tail unpaired t-Student test using the GenEx software. Differentially expressed miRNAs (DEMs) in the Lin-CD34-CD38- cell population were selected as the miRNAs with a Relative Quantity (RQ) ≥ 2 or ≤ 0.5 (*p* < 0.05) in the comparison between CML Lin-CD34-CD38− and Normal donor Lin-CD34-CD38- samples. DEMs in the Lin-CD34+CD38− cell population were selected as the miRNAs with a Relative Quantity (RQ) ≥ 2 or ≤ 0.5 (*p* < 0.05) in the comparison between CML Lin-CD34+CD38− and Normal donor Lin-CD34+CD38− samples. miEP data have been deposited in the GEO public database [34] (http://www.ncbi.nlm.nih.gov/geo; series GSE90773).

### Quantitative reverse transcription polymerase chain reaction (qRT-PCR)

Total RNA (100 ng) was reverse-transcribed to cDNA using a High Capacity cDNA Archive Kit (Life technologies; Carlsbad, CA, USA). TaqMan PCR was carried out using the TaqMan Fast Advanced PCR master mix and TaqMan gene expression assays (all reagents from Life Technologies), by means of a 7900HT Fast Real-Time PCR System (Applied Biosystems). Assays were performed in triplicate. Gene expression profiling was achieved using the comparative cycle threshold (CT) method of relative quantitation using Glyceraldehyde-3-phosphate dehydrogenase (GAPDH) as housekeeping genes. To normalize data, ΔΔCT was calculated for each sample using the mean of its ΔCT values subtracted from the mean ΔCT value measured in the control sample, set as a calibrator; relative quantitation (RQ) value was expressed as 2^−ΔΔCT^.

Individual miRNA detection by RT-qPCR was performed using the TaqMan MicroRNA assays (Life technologies). Individual reverse transcription reactions (5 ng of total RNA each target) were performed using the Taqman microRNA Reverse Transcription Kit and the miRNA-specific looped-primers. TaqMan PCR was performed in triplicate by using the 7900HT Fast Real-Time PCR System (Applied Biosystems). miRNA expression RQ data were calculated as reported above, using U6 snRNA as housekeeping control.

### Electroporation of K562 cells

K562 cells were electroporated by means of the Amaxa 4D-Nucleofector^™^ System, according to the manufacturer's instructions. Briefly, K562 cells were subcultured at a density of 3 × 10^5^ cells/mL 2 days before nucleofection in Iscove's-modified Dulbecco medium (IMDM; Euroclone) supplemented with 10% FBS (Sigma-Aldrich). Each sample was electroporated two times once every 24 hours (h) with 3 μg of mirVana^™^ miR-29a-3p mimic or mirVana^™^ miR-494-3p mimic or mirVana^™^ miR-660-5p mimic or mirVana^™^ miRNA mimic Negative Control #1 (Neg CTR mimic) (Supplementary Table 4) (all from Life Technologies). For each electroporation, 10^6^ cells were resuspended in 100 μL of SF Cell line Solution (Lonza) and pulsed with the program FF120. For gene silencing experiments, each sample was electroporated two times once every 24 hours (h) with 5 μg of Silencer Select small interfering RNA (siRNA) targeting human EPAS1, TET2 or MYC. To exclude non-specific effects caused by interfering RNA (RNAi) nucleofection, a sample transfected with a non-targeting siRNA (NT siRNA; Silencer Select Negative Control #2 siRNA; Life Technologies) (Supplementary Table 4) was included. After each transfection, K562 cells were transferred into pre-warmed fresh medium in 12-well plates and maintained in the same culture conditions as described above. 24 h after last nucleofection, cells were treated with IM 1 μM or 0.4 μM (Sigma-Aldrich), or Dasatinb 100 nM or 25 nM (Bristol-Myers Squibb) or Ponatinib 10 nM or 2,5 nM (Ariad Pharmaceuticals) for additional 24 and 48 h respectively.

For 3′UTR luciferase reporter assays, cells were transiently co-nucleofected with either a miRNA mimic or miRNA mimic negative control (Neg CTR mimic) at a concentration of 3.6 μM and with either 3′UTR-less luciferase or a full-length 3′UTR construct at a concentration of 200 ng/sample. For each electroporation, 10^6^ cells were resuspended in 100 μL of SF Cell line Solution (Lonza) and pulsed with the program FF120.

### CrkL phosphorylation assay

The CrkL Tyr207-phosphorylation assay was performed in untreated and IM-treated K562 cells after either 24h or 48h of IM treatment. For each datapoint, 10^5^ cells per sample were labeled for PCrkL as already described [35]. Cells were analyzed by BD FACSCanto II (BD Biosciences; San Jose, CA USA). At least 10000 events were counted for each sample to ensure statistical relevance. The resulting flow cytometry data were normalized according to the mean fluorescent intensity (MIF) so that the untreated control K562 cell population for each sample had pCrkl MIF = 1. Then, the results for each sample were calculated as a fraction of this.

### Annexin V/PI staining

Apoptosis was evaluated by Annexin V assay (Annexin V-FITC Kit, Trevigen Inc.) as previously described [36]. Briefly, 5 × 10^5^ cells were washed with cold PBS and incubated in 100 μL Annexin V Incubation Reagent for 15 min at room temperature in the dark. After staining, cells were analyzed by using a BD FACSCanto II (BD Biosciences; San Jose, CA USA). At least 100,000 events were counted for each sample to ensure statistical relevance. Apoptotic cells are Annexin V bright and Propidium Iodide (PI) low, late apoptotic cells or necrotic cells are Annexin V and PI bright [37].

### 3′UTR luciferase reporter assays

Empty luciferase reporter constructs (pEZX-MT01) or plasmids containing wild-type full-length 3′UTR from human TET2 (RefSeq NM_001127208.2), human c-MYC (RefSeq NM_002467.4) and human EPAS1 (RefSeq NM_001430.4) transcripts were all purchased from Labomics (Genecopoeia; MD, USA). Luciferase assay and its following normalization were performed as previously reported [12].

### Western blot

Protein levels were assessed by means of Western Blot Analysis in K562 cells (ATCC) overexpressing either miR-29a-3p, miR-660-5p or miR-494-3p. Briefly, cells were harvested 48 hours after the last nucleofection, washed twice with cold phosphate-buffered saline (PBS) and lysed in 50 mM Tris (tris(hydroxymethyl) aminomethane)-Cl (pH 7.4), 150 mM NaCl, 1% Nonidet P-40, 10 mM KCl, 1 mM EDTA, 20 mM NaF, 0.25% Na doexycholate, 5 mM dithiothreitol (DTT). Protease inhibitors (Roche, Indianapolis, IN, USA Complete, catalog #1697498) and phosphatase inhibitors (ThermoFisher Scientific) were added to the lysis buffer. Total cellular lysates (50 μg for each sample) were loaded and separated on 10% SDS-polyacrylamide gel and then transferred on a nitrocellulose membrane. To visualize loading and transfer, Ponceau staining has been performed. Membranes were then pre-blocked in a blocking solution of tris-buffered saline (TBS) containing 5% non-fat dry milk (NFDM) and then incubated with the following primary antibodies: mouse monoclonal anti-TET2 antibody (SantaCruz Biotechnology, Inc, Dallas, Texas, USA; catalog# sc-398535, 1:100 dilution at 4°C overnight), rabbit monoclonal anti-MYC antibody (Abcam; catalog #ab32072; 1:200 dilution at 4°C overnight), rabbit polyclonal anti-EPAS1 antibody (Novus Biological, catalog #NB100-122; 1:500 dilution at 4°C overnight), and with rabbit polyclonal anti-β-actin primary antibody (Thermo Fisher Scientific Inc, catalog #PA1-16889; 1:2000 dilution for 1 hour at RT). The blots were washed for three times with TBS and then incubated with 1:5000 dilution of horseradish peroxidase (HRP) conjugated goat anti-mouse (Santa Cruz Biotechnology, Santa Cruz, CA, USA catalog #sc-2005) and/or 1:1000 dilution of HRP-conjugated goat anti-rabbit secondary antibody (Thermo Fisher Scientific Inc., catalog #32460) for 1 h at RT secondary antibodies. After three successive washes with TBS, BM chemiluminescence Blotting Substrate (POD) (Roche) was used for protein detection.

### CML hematopoietic stem/progenitor cells' isolation and culture

Lineage negative cells from 5 CML patients (Supplementary Table 1) were isolated by means of a custom-made Easy Sep Negative Selection Cocktail (StemCell Technologies, Canada). Lin- cells were seeded at 5 × 10^5^ cells/mL in serum-free medium SYN-H (ABCell-Bio, Paris, France), supplemented with stem cell factor (SCF; 50 ng/mL), Fms-like tyrosine kinase 3 ligand (Flt3L; 50 ng/mL), thrombopoietin (TPO; 20 ng/mL), interleukin-6 (IL-6; 10 ng/mL), and interleukin-3 (IL-3; 10 ng/mL; all from Miltenyi). Lin- cells were transfected using the 4D-Nucleofector^™^ System (Lonza) as previously reported [36]. 24 h after last nucleofection cells were treated with IM 10 μM or 5 μM (Sigma-Aldrich) for 24 h and 48 h respectively, or 200 nM Dasatinib (Bristol-Myers Squibb) or 20 nM Ponatinib (Ariad Pharmaceuticals) for additional 48 h.

### Statistical analysis

The statistics used for data analysis in overexpression/silencing experiments and 3′UTR luciferase reporter assays were based on one-way ANOVA and 2-tailed Student *t*-tests for average comparisons in paired samples (equal variance). Data were analyzed with Microsoft Excel (Microsoft Office, 2011 release) and StatPlus:mac LE (AnalystSoft) and are reported as mean ± standard error of the mean (SEM). A *p*-value < 0.05 was considered significant. Supplementary Table 7.

## SUPPLEMENTARY MATERIALS FIGURES AND TABLES







## Abbreviations

MIF: indicates mean fluorescence intensity
IM: Imatinib Mesylate
NT: Not Treated
NT siRNA: Non-targeting siRNA
siRNA: small interfering RNA
24 h: 24 hours
36 h: 36 hours
48 h: 48 hours
PI: Propidium Iodide
