# Supplementary material for: Deregulated expression of miR-29a-3p, miR-494-3p and miR-660-5p affects sensitivity to tyrosine kinase inhibitors in CML leukemic stem cells
Source: Oncotarget. 2017 May 8;8(30):49451–69. doi: 10.18632/oncotarget.17706 (PMC5564781; doi:10.18632/oncotarget.17706)
Supplement: Supplementary file 1 [file oncotarget-08-49451-s001.pdf]

Deregulated expression of miR-29a-3p, miR-494-3p and miR-660-5p affects sensitivity to tyrosine kinase inhibitors in CML leukemic stem cells

SUPPLEMENTARY MATERIALS

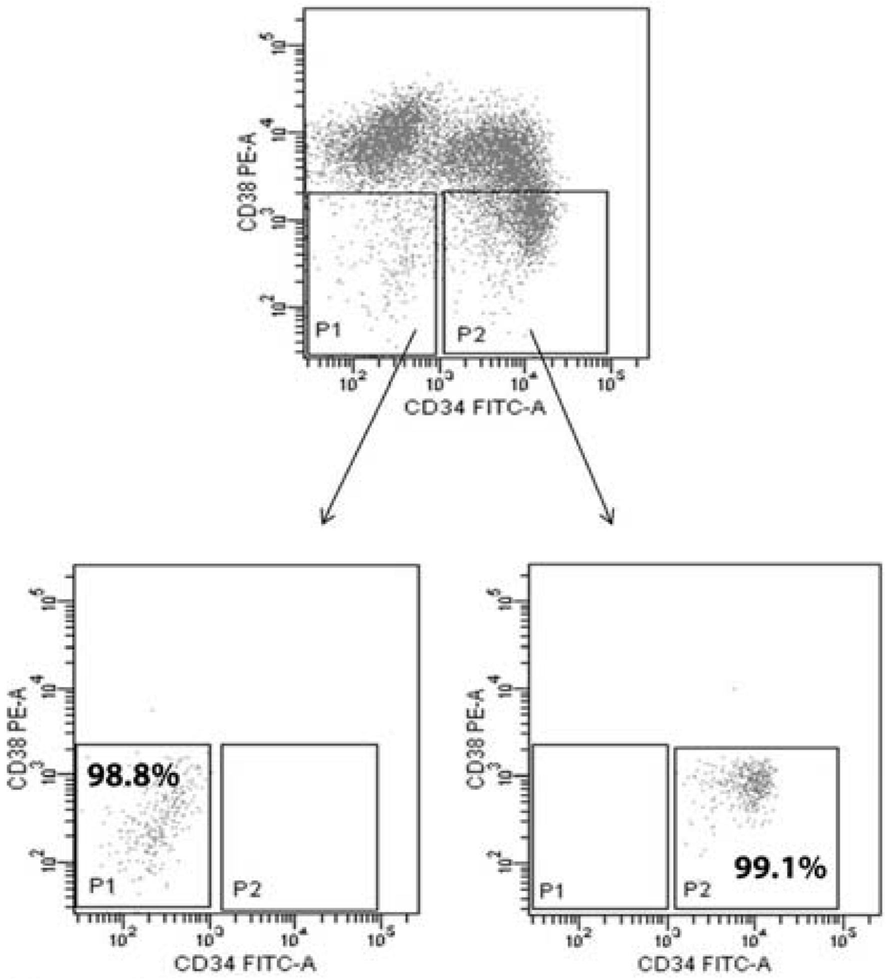

Supplementary Figure 1: Flow cytometric analysis of purified Lin-CD34-CD38- and Lin-CD34+CD38−. Representative dot plot for CML Lin−CD34−CD38− and Lin−CD34+CD38− isolation and reanalysis by FACScan (Becton Dickinson).

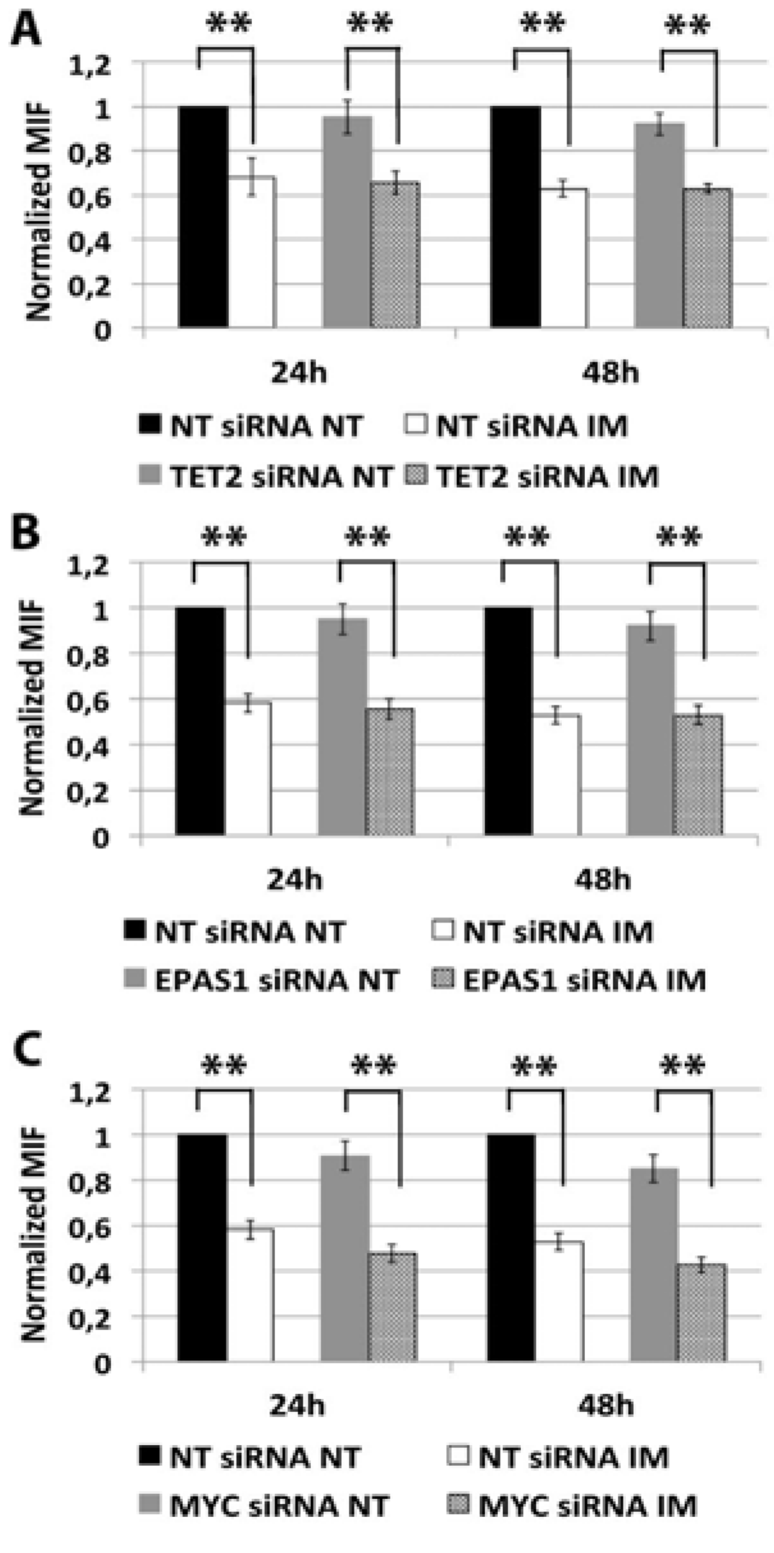

Supplementary Figure 2: Flow cytometric evaluation of pCRKL intracellular level in K562 cells after gene silencing. (**A**) Normalized MIF of intracellular p-CrkL levels in K562 cells after TET2 silencing. pCRKL MIF of the NT siRNA untreated control was set to 1 to compare K562 before and after treatment with IM. The results are expressed as mean values ± SEM ($n = 3$). (**B**) Normalized MIF of intracellular p-CrkL levels in K562 cells after EPAS1 silencing. pCRKL MIF of the NT siRNA untreated control was set to 1 to compare K562 before and after treatment with IM. The results are expressed as mean values ± SEM ($n = 3$). (**C**) Normalized MIF of intracellular p-CrkL levels in K562 cells after MYC silencing. pCRKL MIF of the NT siRNA untreated control was set to 1 to compare K562 before and after treatment with IM. The results are expressed as mean values ± SEM ($n = 3$). **$p < 0.01$ in untreated versus IM-treated. Abbreviations: MIF indicates Mean Fluorescence Intensity; IM, Imatinib Mesylate; NT siRNA, Non-targeting siRNA; siRNA, small interfering RNA; NT, Not Treated; 24 h, 24 hours; 48 h, 48 hours.

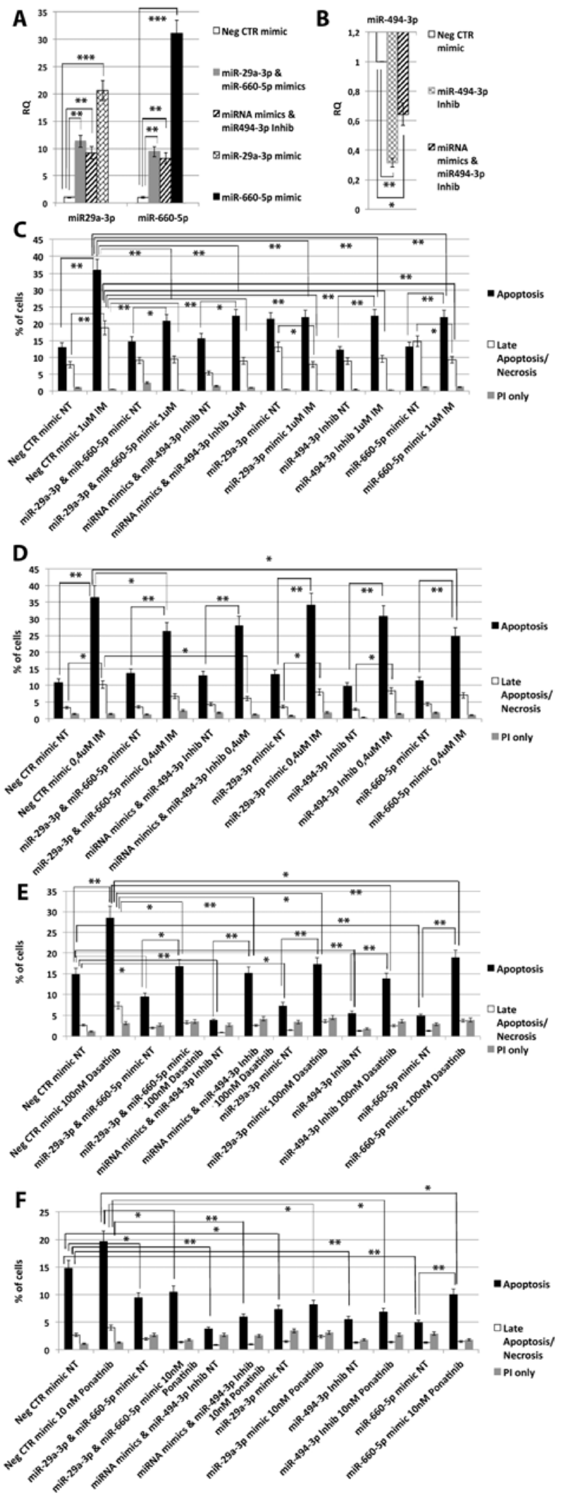

Supplementary Figure 3: Effects of combined miR-29a-3p and miR-660-5p overexpression and miR-494-3p downregulation on K562 cells' response to TKIs. (**A**) Expression levels of miR-29a-3p and miR-660-5p 24 hours after the last nucleofection of the respective miRNA mimics as evaluated by qRT-PCR. Data are reported as RQ mean ± S.E.M of 3 independent experiments. (**B**) Expression levels of miR-494-3p 24 hours after the last nucleofection of the miR-494-3p Inhibitor as evaluated by qRT-PCR. Data are reported as RQ mean ± S.E.M of 3 independent experiments. Results of Annexin V/PI staining on K562 cells after 24 h (**C**) and 48 h (**D**) of IM treatment (mean±SEM; $n = 3$). (**E**) Results of Annexin V/PI staining on K562 cells after 24 h of Dasatinib treatment (mean ± SEM; $n = 3$). (**F**) Results of Annexin V/PI staining on K562 cells after 24h of Ponatinib treatment (mean ± SEM; $n = 3$) $*p < 0.05$, $**p < 0.01$. Apoptotic cells (black bars in the histogram plot) are Annexin V bright and PI low, late apoptotic cells or necrotic cells (white bars in the histogram plot) are Annexin V and PI bright, grey bars represent cells bright for PI only. Abbreviations: RQ, Relative Quantity; MIF indicates Mean Fluorescence Intensity; IM, Imatinib Mesylate; NT, Not Treated; 24 h, 24 hours; 48 h, 48 hours; PI, Propidium Iodide; miRNA mimics: miR-29a-3p mimic and miR-660-5p mimic.

MATERIALS AND METHODS

Electroporation of K562 cells with miRNA mimics and miRNA inhibitor

K562 cells were electroporated by means of the Amaxa 4D-Nucleofector™ System, according to the manufacturer's instructions. Briefly, K562 cells were subcultured at a density of 3×10^5 cells/mL 2 days before nucleofection in Iscove's-modified Dulbecco medium (IMDM; Euroclone) supplemented with 10% FBS (Sigma-Aldrich). Each sample was electroporated two times once every 24 hours (h) with 3 µg of mirVana™ miR-29a-3p mimic or mirVana™ miR-494-3p mimic or mirVana™ miR-660-5p mimic or mirVana™ miRNA mimic Negative Control #1 (Neg CTR mimic) (all from Life Technologies). For miRNA inhibition experiments, K562 cells were nucleofected twice using the same pulsing protocol, once every 24 hours, with 1 µg (0,21 nmol) of in vivo LNA™ miR-494-3p inhibitor or the same quantity of a scramble negative control inhibitor (Exiqon). To assess the combined effect of miR-29a-3p and miR-660-5p, K562 cells were nucleofected twice with 1,5 µg of each miRNA mimic. To assess the combined effect of miR-29a-3p and miR-660-5p overexpression together with miR-494-3p inhibition, K562 cells were nucleofected twice with 1 µg of each miRNA mimic and 0,5 µg of miR-494-3p Inhibitor. For each electroporation, 10^6 cells were resuspended in 100 µL of SF Cell line Solution (Lonza) and pulsed with the program FF120. After each transfection, K562 cells were transferred into pre-warmed fresh medium in 12-well plates and maintained in the same culture conditions as described above. 24 h after last nucleofection, cells were treated with Imatinib 1 µM or 0,4 µM (Sigma-Aldrich), or Dasatinib 100 nM or 25 nM (Bristol-Myers Squibb) or Ponatinib 10 nM or 2,5 nM (Ariad Pharmaceuticals) for additional 24 and 48 h respectively.

Supplementary Table 1: Patient characteristics

miRNA expression profiling

# PT	Gender	Age	Sokal Risk	CCA Ph+	Transcript type
1	F	62	High	No	b3a2
2	F	49	Intermediate	No	b3a2
3	M	41	Intermediate	No	b2a2
4	M	74	Intermediate	No	b3a2
5	M	71	Intermediate	45,X,-Y,t(9;22)(q34;q11)	b2a2

miRNA overexpression experiments

# PT	Gender	Age	Sokal Risk	CCA Ph+	Transcript type
6	F	65	Intermediate	No	b2a2
7	M	71	Intermediate	No	b2a2
8	F	49	Low	No	b3a2
9	F	57	High	No	b2a2
10	F	50	Intermediate	No	b3a2

Legend: CCA Ph+: additional Clonal Cytogenetic Abnormalities in Philadelphia positive cells.

Supplementary Table 2: Deregulated miRNAs in the comparison CML Lin-CD34-CD38- vs Normal Donor Lin-CD34-CD38−. See Supplementary_Table_2

Supplementary Table 3: Deregulated miRNAs in the comparison CML Lin-CD34+CD38− vs Normal Donor Lin-CD34+CD38−. See Supplementary_Table_3

Supplementary Table 4: List of miRNA mimics and siRNAs

miRNA/gene ID	mimic/siRNA ID (Life Technologies)
hsa-miR-29a-3p	MC12499
hsa-miR-494-3p	MC12409
hsa-miR-660-5p	MC11216
TET2	s29442
MYC	s9130
EPAS1	s4698
miRNA Inhibitor	In vivo LNA™ microRNA Inhibitors (Exiqon)
hsa-miR-494-3p miRCURY LNA™ microRNA inhibitor	4103042-001

Supplementary Table 5: List of deregulated miRNAs in the comparison CML Lin-CD34-CD38- vs normal Lin-CD34-CD38- with FDR

miRNA ID	FC CML Lin-CD34-CD38- vs Normal Lin-CD34-CD38-	*q*-value
hsa-miR-188-3p	76,45294	0,01179
hsa-miR-486-5p	−25,30762	0,01179
hsa-miR-532-3p	17,63048	0,01179
hsa-miR-29a-3p	6,11775	0,01179
hsa-miR-501-5p	11,8392	0,01179
hsa-miR-1207-5p	31,87824	0,01179
hsa-miR-660-5p	16,08339	0,01608
hsa-miR-193b-5p	24,4625	0,02537
hsa-miR-193b-3p	65,70813	0,0432
hsa-miR-204-5p	−19,09337	0,0432
hsa-miR-532-5p	12,62753	0,0432
hsa-miR-134	−4,40457	0,0432
hsa-miR-362-3p	22,61174	0,0432
hsa-miR-874	2,78466	0,0443
hsa-miR-362-5p	8,90429	0,04671
hsa-miR-502-3p	13,12732	0,04671

Supplementary Table 6: List of deregulated miRNAs in the comparison CML Lin-CD34+CD38- vs normal Lin-CD34+CD38- with FDR

miRNA ID	FC CML Lin-CD34+CD38- vs Normal Lin-CD34+CD38-	*q*-value
hsa-miR-324-5p	2,55974	0,00902
hsa-miR-32-5p	6,51606	0,04141
hsa-miR-486-3p	−26,45558	0,04141
hsa-miR-22-3p	7,52139	0,04141
hsa-miR-631	−5,90526	0,04141
hsa-miR-660-5p	12,19316	0,04345
hsa-miR-106b-5p	2,11991	0,04345
hsa-miR-362-3p	11,59154	0,04345
hsa-miR-15a-5p	2,76638	0,04345
hsa-miR-21-5p	9,7001	0,04345
hsa-miR-33a-5p	5,11772	0,04345
hsa-miR-141-5p	−7,75432	0,04345

Supplementary Table 7: Results of one-way ANOVA analysis on miRNA over-expression experiments and siRNA-mediated gene silencing experiments in K562 cells

K562 cell	one-way ANOVA			
**Apoptosis**	***p*-value IM 24 h**	***p*-value IM 48 h**	***p*-value Ponatinib 24 h**	***p*-value Dasatinib 24 h**
miR-29a-3p mimic	0.00443	0.01067	0.00341	0.00914
miR-494-3p mimic	0.00131	0.00048	0.00152	0.00276
miR-660-5p mimic	0.00818	0.00201	0.00483	0.00259
**Late apoptosis/Necrosis**	***p*-value IM 24 h**	***p*-value IM 48 h**	***p*-value Ponatinib 24 h**	***p*-value Dasatinib 24 h**
miR-29a-3p mimic	0.00251	0.00476	0.06851	0.04238
miR-494-3p mimic	0,00000386	0.00021	0.08952	0.03897
miR-660-5p mimic	0.00847	0.00318	0.07472	0.06539

K562 cell	one-way ANOVA	
**Apoptosis**	***p*-value IM 24 h**	***p*-value IM 48 h**
TET2 siRNA	0.04442	0.01715
MYC siRNA	0.00202	0.00089
EPAS1 siRNA	0.03417	1,27E-08
**Late apoptosis/Necrosis**	***p*-value IM 24 h**	***p*-value IM 48 h**
TET2 siRNA	0.04561	0.00058
MYC siRNA	0.00398	0.00001
EPAS1 siRNA	0.04238	0.00009

Supplementary Table 8: Results of one-way ANOVA analysis on miRNA over-expression experiments in CML Lin-CD34-CD38- cells

CML CD34-CD38-	one-way ANOVA		
**Apoptosis**	***p*-value IM 48 h**	***p*-value Ponatinib 48 h**	***p*-value Dasatinib 48 h**
miR-29a-3p mimic	0.01076	0.02671	0.01803
miR-494-3p mimic	0.00503	0.00612	0.00575
miR-660-5p mimic	0.01141	0.02194	0.03178
**Late apoptosis/Necrosis**	***p*-value IM 48 h**	***p*-value Ponatinib 48 h**	***p*-value Dasatinib 48 h**
miR-29a-3p mimic	0.00002	0.00097	0.00034
miR-494-3p mimic	1,04E-06	0.00053	0.00028
miR-660-5p mimic	0.00020	0.00049	0.00031

Supplementary Table 9: Results of one-way ANOVA analysis on miRNA over-expression experiments in CML Lin-CD34+CD38− cells

CML CD34+CD38-	one-way ANOVA		
**Apoptosis**	***p*-value IM 48 h**	***p*-value Ponatinib 48 h**	***p*-value Dasatinib 48 h**
miR-29a-3p mimic	0.01811	0.02713	0.31771
miR-494-3p mimic	0.02439	0.01965	0.02518
miR-660-5p mimic	0.03232	0.02986	0.04059
Late apoptosis/Necrosis	*p*-value IM 48 h	*p*-value Ponatinib 48 h	*p*-value Dasatinib 48 h
miR-29a-3p mimic	6,40E-08	0.00017	0.00023
miR-494-3p mimic	0.00006	0.00009	0.00002
miR-660-5p mimic	5,09E-10	1,20E-06	5,30E-08
