# Supplementary material for: Deregulated expression of miR-29a-3p, miR-494-3p and miR-660-5p affects sensitivity to tyrosine kinase inhibitors in CML leukemic stem cells
Source: Oncotarget. 2017 May 8;8(30):49451–69. doi: 10.18632/oncotarget.17706 (PMC5564781; doi:10.18632/oncotarget.17706)
Supplement: Supplementary file 2 [file oncotarget-08-49451-s002.docx]

**Table S2.** Deregulated miRNAs in the comparison CML Lin-CD34-CD38- vs Normal Donor Lin-CD34-CD38-.

| **miRNA ID** | **FC CML Lin-CD34-CD38- vs Normal Lin-CD34-CD38-** | **P-Value CML Lin-CD34-CD38- vs Normal Lin-CD34-CD38-** |
| --- | --- | --- |
| hsa-miR-188-3p | 76,45294 | 2,86E-05 |
| hsa-miR-486-5p | -25,30762 | 4,44E-05 |
| hsa-miR-532-3p | 17,63048 | 7,34E-05 |
| hsa-miR-29a-3p | 6,11775 | 7,68E-05 |
| hsa-miR-501-5p | 11,8392 | 9,98E-05 |
| hsa-miR-1207-5p | 31,87824 | 0,000108854 |
| hsa-miR-660-5p | 16,08339 | 0,000173131 |
| hsa-miR-193b-5p | 24,4625 | 0,000312233 |
| hsa-miR-193b-3p | 65,70813 | 0,000687716 |
| hsa-miR-204-5p | -19,09337 | 0,000717976 |
| hsa-miR-532-5p | 12,62753 | 0,000840291 |
| hsa-miR-134 | -4,40457 | 0,000855181 |
| hsa-miR-362-3p | 22,61174 | 0,00086403 |
| hsa-miR-874 | 2,78466 | 0,000954076 |
| hsa-miR-362-5p | 8,90429 | 0,001082308 |
| hsa-miR-502-3p | 13,12732 | 0,001149869 |
| hsa-miR-585 | -4,40915 | 0,001405014 |
| hsa-miR-487a | -4,24422 | 0,001417284 |
| hsa-miR-29a-5p | 8,70896 | 0,001516468 |
| hsa-let-7g-5p | 2,26655 | 0,001855048 |
| hsa-miR-29c-3p | 2,59998 | 0,001942691 |
| hsa-miR-182-5p | -10,53779 | 0,002201963 |
| hsa-miR-486-3p | -14,21163 | 0,002514497 |
| hsa-miR-652-3p | -3,17664 | 0,002757243 |
| hsa-miR-186-5p | 2,84711 | 0,003153779 |
| hsa-miR-567 | -3,30351 | 0,003581082 |
| hsa-miR-181c-5p | 3,89737 | 0,00391365 |
| hsa-miR-95 | 23,79357 | 0,004569115 |
| hsa-miR-500a-5p | 11,45972 | 0,00519193 |
| hsa-miR-29b-3p | 3,86241 | 0,005216321 |
| hsa-miR-200a-3p | 13,73705 | 0,005411673 |
| hsa-miR-187-3p | 13,22321 | 0,006324648 |
| hsa-miR-342-3p | 4,69622 | 0,006502927 |
| hsa-miR-130a-5p | -3,43426 | 0,007714394 |
| hsa-miR-188-5p | 26,71355 | 0,007758605 |
| hsa-miR-610 | -2,64085 | 0,007844752 |
| hsa-miR-223-3p | -3,21318 | 0,00811193 |
| hsa-miR-501-3p | 34,22551 | 0,008250211 |
| hsa-miR-605 | -3,85706 | 0,008513337 |
| hsa-miR-342-5p | 5,99392 | 0,009091031 |
| hsa-miR-485-3p | -14,81514 | 0,009264518 |
| hsa-miR-551b-3p | 7,02501 | 0,009350089 |
| hsa-miR-99a-5p | 3,31843 | 0,009904307 |
| hsa-miR-363-3p | 2,14132 | 0,010381868 |
| hsa-miR-1539 | -3,36825 | 0,010885734 |
| hsa-miR-382-3p | -5,20717 | 0,010937939 |
| hsa-miR-103b | -2,81376 | 0,010959171 |
| hsa-miR-515-3p | -2,81084 | 0,01146872 |
| hsa-miR-1200 | -2,81084 | 0,01146872 |
| hsa-miR-630 | -2,99488 | 0,011838255 |
| hsa-miR-675-5p | -2,80597 | 0,012078421 |
| hsa-miR-708-5p | 26,52903 | 0,012839647 |
| hsa-miR-519e-5p | -2,77695 | 0,014546851 |
| hsa-miR-145-5p | -5,80582 | 0,014938507 |
| hsa-miR-889 | -3,27728 | 0,015250287 |
| hsa-miR-520h | -3,18656 | 0,015302417 |
| hsa-let-7i-5p | 2,09217 | 0,01646008 |
| hsa-miR-452-5p | -2,54824 | 0,016460711 |
| hsa-miR-219-2-3p | -2,58023 | 0,017762685 |
| hsa-miR-593-5p | -2,66567 | 0,017792805 |
| hsa-miR-146b-5p | 2,82647 | 0,018246594 |
| hsa-miR-1272 | -4,12387 | 0,01888584 |
| hsa-miR-518f-3p | -2,42503 | 0,019107856 |
| hsa-miR-2110 | 2,87687 | 0,019259448 |
| hsa-miR-494 | -2,63171 | 0,019666384 |
| hsa-miR-548i | -2,75108 | 0,019710068 |
| hsa-miR-10b-3p | -2,43598 | 0,020147356 |
| hsa-miR-645 | -2,74632 | 0,020493746 |
| hsa-miR-23b-5p | -5,35728 | 0,020859019 |
| hsa-miR-765 | -2,49234 | 0,02124451 |
| hsa-miR-200a-5p | -2,83726 | 0,022267976 |
| hsa-miR-302e | -2,70288 | 0,022534839 |
| hsa-miR-449a | -3,66293 | 0,022766277 |
| hsa-miR-622 | -2,86295 | 0,023461742 |
| hsa-miR-626 | -3,02828 | 0,023502448 |
| hsa-miR-767-5p | -2,71791 | 0,023607604 |
| hsa-miR-518d-3p | -2,71791 | 0,023607604 |
| hsa-miR-181b-5p | 2,04982 | 0,023738914 |
| hsa-miR-206 | -7,34366 | 0,023884673 |
| hsa-miR-875-3p | -2,3473 | 0,024082004 |
| hsa-miR-155-5p | 2,36035 | 0,024409326 |
| hsa-miR-625-3p | -14,05002 | 0,024482997 |
| hsa-miR-1184 | -2,21453 | 0,024493397 |
| hsa-miR-199a-3p | -2,44274 | 0,024543652 |
| hsa-miR-221-3p | -2,26734 | 0,024863762 |
| hsa-miR-19b-2-5p | -3,31153 | 0,024893794 |
| hsa-miR-330-5p | 3,15032 | 0,025518378 |
| hsa-miR-365a-3p | 19,3532 | 0,025761122 |
| hsa-miR-587 | -2,59998 | 0,025906995 |
| hsa-miR-224-3p | -2,59998 | 0,025906995 |
| hsa-miR-609 | -2,80208 | 0,027003981 |
| hsa-miR-1204 | -2,51403 | 0,027212345 |
| hsa-miR-142-3p | 3,52592 | 0,027214544 |
| hsa-miR-150-5p | 12,22278 | 0,027401201 |
| hsa-miR-302c-5p | -3,7607 | 0,028415698 |
| hsa-miR-518e-5p | -2,32302 | 0,028932782 |
| hsa-miR-601 | -2,45207 | 0,029091885 |
| hsa-miR-450b-3p | -2,47942 | 0,029820778 |
| hsa-miR-376b-3p | -8,42689 | 0,030215319 |
| hsa-miR-33b-3p | -7,33095 | 0,030410462 |
| hsa-miR-125b-5p | 2,53327 | 0,031481492 |
| hsa-miR-22-3p | 3,54307 | 0,032041778 |
| hsa-miR-409-5p | -4,40457 | 0,032778115 |
| hsa-miR-655 | -2,46314 | 0,033864161 |
| hsa-miR-517-5p | -2,77214 | 0,034002064 |
| hsa-miR-603 | -2,59278 | 0,034337056 |
| hsa-miR-140-5p | 2,17272 | 0,034660318 |
| hsa-miR-155-3p | 3,49671 | 0,034841392 |
| hsa-miR-554 | -2,35137 | 0,034874632 |
| hsa-miR-541-5p | -2,76734 | 0,034999591 |
| hsa-miR-518f-5p | -2,21991 | 0,035360908 |
| hsa-miR-24-1-5p | 3,29436 | 0,035373702 |
| hsa-miR-634 | -2,77695 | 0,035844467 |
| hsa-miR-490-5p | -6,20315 | 0,037706044 |
| hsa-miR-448 | -2,40744 | 0,039415055 |
| hsa-miR-381-5p | -4,18434 | 0,039548966 |
| hsa-miR-105-3p | -2,38006 | 0,039594297 |
| hsa-miR-888-3p | -2,13022 | 0,039719512 |
| hsa-miR-541-3p | -2,27363 | 0,040224476 |
| hsa-miR-194-3p | 3,47256 | 0,041073674 |
| hsa-miR-1247-5p | -2,37347 | 0,042004795 |
| hsa-miR-298 | -2,61897 | 0,042631431 |
| hsa-miR-135a-3p | -4,71416 | 0,042702883 |
| hsa-miR-1909-3p | -2,38171 | 0,04422185 |
| hsa-miR-1179 | -3,07695 | 0,044960347 |
| hsa-miR-455-5p | -2,13613 | 0,045740031 |
| hsa-miR-548c-5p | -2,77791 | 0,046540028 |
| hsa-miR-490-3p | -3,73343 | 0,04752617 |
| hsa-miR-21-5p | 3,07801 | 0,047679062 |
| hsa-miR-2113 | -6,71785 | 0,049541708 |
| hsa-miR-548m | -2,38171 | 0,049610259 |
| hsa-miR-1269a | -2,38171 | 0,049610259 |
| hsa-miR-518e-3p | -2,09943 | 0,049685053 |
| hsa-miR-584-5p | -5,39267 | 0,049934999 |
