# Supplementary material for: Deregulated expression of miR-29a-3p, miR-494-3p and miR-660-5p affects sensitivity to tyrosine kinase inhibitors in CML leukemic stem cells
Source: Oncotarget. 2017 May 8;8(30):49451–69. doi: 10.18632/oncotarget.17706 (PMC5564781; doi:10.18632/oncotarget.17706)
Supplement: Supplementary file 3 [file oncotarget-08-49451-s003.docx]

**Table S3**. Deregulated miRNAs in the comparison CML Lin-CD34+CD38- vs Normal Donor Lin-CD34+CD38-.

| **miRNA ID** | **FC CML Lin-CD34+CD38- vs Normal Lin-CD34+CD38-** | **P-Value CML Lin-CD34+CD38- vs Normal Lin-CD34+CD38-** |
| --- | --- | --- |
| hsa-miR-708-5p | 74,18214 | 0,00174831 |
| hsa-miR-193b-3p | 61,54202 | 0,002720273 |
| hsa-miR-21-3p | 14,76388 | 0,002431931 |
| hsa-miR-188-5p | 14,38008 | 0,005384551 |
| hsa-miR-9-5p | 14,22641 | 0,005940888 |
| hsa-miR-660-5p | 12,19316 | 0,000451406 |
| hsa-miR-362-3p | 11,59154 | 0,000634249 |
| hsa-miR-1537 | 10,31377 | 0,005514904 |
| hsa-miR-21-5p | 9,7001 | 0,000747818 |
| hsa-miR-1207-5p | 8,58013 | 0,009123468 |
| hsa-miR-451a | 8,52677 | 0,04347118 |
| hsa-miR-22-3p | 7,52139 | 0,000303853 |
| hsa-miR-141-3p | 7,39474 | 0,035160195 |
| hsa-miR-32-5p | 6,51606 | 0,000140878 |
| hsa-miR-150-5p | 5,57704 | 0,003257578 |
| hsa-miR-142-3p | 5,15153 | 0,003748156 |
| hsa-miR-33a-5p | 5,11772 | 0,000751361 |
| hsa-miR-362-5p | 4,80488 | 0,004675679 |
| hsa-miR-590-5p | 4,75683 | 0,001326578 |
| hsa-miR-500a-5p | 4,75683 | 0,027109318 |
| hsa-miR-532-5p | 4,55989 | 0,002801071 |
| hsa-miR-502-3p | 4,54254 | 0,005614334 |
| hsa-miR-548k | 4,52997 | 0,027429796 |
| hsa-miR-545-3p | 4,40151 | 0,034635561 |
| hsa-miR-532-3p | 4,39542 | 0,007333815 |
| hsa-miR-301a-3p | 4,3334 | 0,007679798 |
| hsa-miR-142-5p | 4,10249 | 0,001221909 |
| hsa-miR-450a-5p | 3,97512 | 0,015815825 |
| hsa-miR-501-3p | 3,605 | 0,019900424 |
| hsa-miR-365a-3p | 3,605 | 0,030619338 |
| hsa-miR-22-5p | 3,20872 | 0,02988847 |
| hsa-miR-140-5p | 3,09728 | 0,005986452 |
| hsa-let-7i-3p | 3,07482 | 0,041993713 |
| hsa-miR-15a-3p | 3,05993 | 0,032601983 |
| hsa-miR-15a-5p | 2,76638 | 0,000700786 |
| hsa-miR-185-5p | 2,6666 | 0,02464642 |
| hsa-miR-941 | 2,64268 | 0,038352311 |
| hsa-miR-590-3p | 2,61716 | 0,04925432 |
| hsa-miR-324-5p | 2,55974 | 1,39E-05 |
| hsa-miR-27a-3p | 2,53415 | 0,044672855 |
| hsa-miR-148b-3p | 2,39164 | 0,002559633 |
| hsa-miR-374a-5p | 2,32221 | 0,022999984 |
| hsa-miR-30e-5p | 2,2839 | 0,029676536 |
| hsa-miR-338-3p | 2,26891 | 0,025925861 |
| hsa-miR-106b-5p | 2,11991 | 0,000510209 |
| hsa-miR-24-2-5p | 2,07556 | 0,015795636 |
| hsa-miR-320b | -2,0223 | 0,00416437 |
| hsa-miR-320c | -2,05979 | 0,012732811 |
| hsa-miR-320d | -2,25324 | 0,008605529 |
| hsa-let-7b-5p | -2,59458 | 0,019735875 |
| hsa-miR-1271-5p | -2,60359 | 0,005931437 |
| hsa-miR-146a-5p | -2,63262 | 0,033929214 |
| hsa-let-7e-5p | -2,67771 | 0,04259122 |
| hsa-miR-92a-3p | -2,70101 | 0,019743252 |
| hsa-miR-369-5p | -2,90895 | 0,007993591 |
| hsa-miR-625-3p | -3,05463 | 0,011005231 |
| hsa-miR-134 | -3,14923 | 0,037523343 |
| hsa-miR-1972 | -3,39756 | 0,026305573 |
| hsa-miR-125b-5p | -3,48945 | 0,042772624 |
| hsa-miR-1244 | -3,74769 | 0,021958345 |
| hsa-miR-376a-5p | -3,99169 | 0,017249173 |
| hsa-miR-519e-3p | -4,03481 | 0,01220945 |
| hsa-miR-155-5p | -4,18579 | 0,007727114 |
| hsa-miR-208a | -4,40457 | 0,027732831 |
| hsa-miR-1267 | -4,61075 | 0,017946439 |
| hsa-miR-875-3p | -4,62355 | 0,028431036 |
| hsa-miR-129-1-3p | -4,70763 | 0,032590424 |
| hsa-miR-647 | -4,70763 | 0,048963358 |
| hsa-miR-593-5p | -4,90754 | 0,010575277 |
| hsa-miR-933 | -4,9864 | 0,003022683 |
| hsa-miR-510 | -5,01066 | 0,010605225 |
| hsa-miR-216a-5p | -5,348 | 0,037748716 |
| hsa-miR-380-3p | -5,53468 | 0,004825 |
| hsa-miR-562 | -5,64706 | 0,035932547 |
| hsa-miR-214-5p | -5,78975 | 0,001326058 |
| hsa-miR-1185-5p | -5,78975 | 0,043038329 |
| hsa-miR-631 | -5,90526 | 0,000318569 |
| hsa-miR-577 | -6,21175 | 0,030175835 |
| hsa-miR-7-2-3p | -6,25496 | 0,013454397 |
| hsa-miR-659-3p | -6,37754 | 0,03870118 |
| hsa-miR-494 | -6,46881 | 0,05088682 |
| hsa-miR-1911-3p | -6,58645 | 0,031442507 |
| hsa-miR-92a-1-5p | -6,72018 | 0,036985161 |
| hsa-miR-573 | -6,75989 | 0,001889749 |
| hsa-miR-204-5p | -6,83528 | 0,024504002 |
| hsa-miR-935 | -7,34876 | 0,012637265 |
| hsa-miR-33b-3p | -7,70877 | 0,025440303 |
| hsa-miR-141-5p | -7,75432 | 0,000802189 |
| hsa-miR-1468 | -7,88713 | 0,024214769 |
| hsa-miR-518d-3p | -7,90355 | 0,025105 |
| hsa-miR-92a-2-5p | -8,38319 | 0,022425814 |
| hsa-miR-25-5p | -8,4415 | 0,013776744 |
| hsa-miR-100-5p | -9,22149 | 0,031207449 |
| hsa-miR-129-2-3p | -9,57319 | 0,018248896 |
| hsa-miR-609 | -10,27809 | 0,030259136 |
| hsa-miR-605 | -10,9359 | 0,008223535 |
| hsa-miR-26a-1-3p | -11,66408 | 0,021554626 |
| hsa-miR-639 | -13,17746 | 0,009972977 |
| hsa-miR-490-5p | -13,82301 | 0,004439018 |
| hsa-miR-432-3p | -14,59093 | 0,01477693 |
| hsa-miR-486-5p | -15,85647 | 0,006116867 |
| hsa-miR-486-3p | -26,45558 | 0,000230935 |
